# Co-occurrence of direct and indirect extracellular electron transfer mechanisms during electroactive respiration in a dissimilatory sulfate reducing bacterium

**DOI:** 10.1128/spectrum.01226-24

**Published:** 2024-12-05

**Authors:** Liyuan Hou, Rebecca Cortez, Michael Hagerman, Zhiqiang Hu, Erica L.-W. Majumder

**Affiliations:** 1 Department of Civil and Environmental Engineering, Utah State University, Logan, Utah, USA; 2 Utah Water Research Laboratory, Logan, Utah, USA; 3 Department of Bacteriology, University of Wisconsin-Madison, Madison, Wisconsin, USA; 4 Department of Mechanical Engineering, Union College, Schenectady, New York, USA; 5 Department of Chemistry, Union College, Schenectady, New York, USA; 6 Department of Civil and Environmental Engineering, University of Missouri, Columbia, Missouri, USA; University of Southern Denmark, Odense, Denmark

**Keywords:** electron transfer mechanisms, *Desulfovibrio vulgaris *Hildenborough, electrically conductive pili, electroactive respiration, microbial fuel cells

## Abstract

Understanding the extracellular electron transfer mechanisms of electroactive bacteria could help determine their potential in microbial fuel cells (MFCs) and their microbial syntrophy with redox-active minerals in natural environments. However, the mechanisms of extracellular electron transfer to electrodes by sulfate-reducing bacteria (SRB) remain underexplored. Here, we utilized double-chamber MFCs with carbon cloth electrodes to investigate the extracellular electron transfer mechanisms of *Desulfovibrio vulgaris* Hildenborough (*Dv*H), a model SRB, under varying lactate and sulfate concentrations using different *Dv*H mutants. Our MFC setup indicated that *Dv*H can harvest electrons from lactate at the anode and transfer them to cathode, where *Dv*H could further utilize these electrons. Patterns in current production compared with variations of electron donor/acceptor ratios in the anode and cathode suggested that attachment of *Dv*H to the electrode and biofilm density were critical for effective electricity generation. Electron microscopy analysis of *Dv*H biofilms indicated *Dv*H utilized filaments that resemble pili to attach to electrodes and facilitate extracellular electron transfer from cell to cell and to the electrode. Proteomics profiling indicated that *Dv*H adapted to electroactive respiration by presenting more pili- and flagellar-related proteins. The mutant with a deletion of the major pilus-producing gene yielded less voltage and far less attachment to both anodic and catholic electrodes, suggesting the importance of pili in extracellular electron transfer. The mutant with a deficiency in biofilm formation, however, did not eliminate current production indicating the existence of indirect extracellular electron transfer. Untargeted metabolomics profiling showed flavin-based metabolites, potential electron shuttles.

**IMPORTANCE:**

We explored the application of *Desulfovibrio vulgaris* Hildenborough in microbial fuel cells (MFCs) and investigated its potential extracellular electron transfer (EET) mechanism. We also conducted untargeted proteomics and metabolomics profiling, offering insights into how DvH adapts metabolically to different electron donors and acceptors. An understanding of the EET mechanism and metabolic flexibility of *Dv*H holds promise for future uses including bioremediation or enhancing efficacy in MFCs for wastewater treatment applications.

## INTRODUCTION

Microbial fuel cells (MFCs) have been used as a promising technology for electrical energy generation, which uses microbes to transfer the chemical energy of organic compounds into electricity (1). Novel insights have been incorporated into MFCs for energy generation as well as the microbial transformation of wastes. For instance, MFCs have been investigated for the conversion of wastewater containing organic compounds and sulfate to electricity using sulfate-reducing bacteria (SRB) and sulfide-oxidizing bacteria (2). These sulfate-containing wastewaters are produced by many processes including mining, food processing, pulp and paper wastewater, animal husbandry, and more (3). By culturing SRB in MFCs, previous studies have achieved the removal of sulfate and organic compounds with electricity production (4, 5), ranging from 0.013 W/m^2^ to 0.68 W/m^2^ (6–8). The overall electricity generation of an MFC mainly relies on the efficiency of extracellular electron transfer (EET) from electrogenic bacteria to the electrode (9).

Electrogenic bacteria can route their electron transport chain to the exterior of the cell through various EET mechanisms (10). Two major EET mechanisms are direct electron transfer and indirect electron transfer. Direct electron transfer mainly relies on outer surface redox molecules, proteins, and conductive nanowires. For instance, in many species, such as *Geobacter sulfurreducens*, *Shewanella oneidensis*, and *Acidithiobacillus ferrooxidans*, EET can be mediated by outer membrane *c*-type cytochromes (e.g., OmcA-MtrCAB protein complexes) (11). *G. sulfurreducens* also exchange electrons through nanowires, which are pili formed by protein filaments (12). *S. oneidensis* MR-1 forms nanowires through extensions of the outer membrane and periplasm that include the multiheme cytochromes that are responsible for EET (13). Indirect electron transfer involves the transfer of electrons through small redox-active organic molecules, electron shuttles, excreted by cells or added exogenously. Different species secrete various extracellular electron carriers such as flavins and phenazine derivatives (14). Previous studies demonstrated the coexistence of direct electron transfer and indirect electron transfer in *S. oneidensis* (15). *S. oneidensis* could simultaneously transfer electrons through direct contact with the electron acceptor and also produce flavins (15). Large numbers of studies have demonstrated that *Geobacter* and *Shewanella* species can power MFCs with high power density (11, 12, 16, 17), due to their unique EET mechanism.

SRB can utilize organic compounds and gases (e.g., hydrogen) as electron donors (18). Recent studies also found that some SRB can use electrodes as electron donors for energy production (19). Despite this, the mechanism for SRB extracellular electron uptake is not clear due to the difficulty in distinguishing the electron uptake reaction (e.g., EET mechanism) and hydrogen evolution on the electrode surface. Knowledge of EET mechanisms has major implications for being able to understand, control, or intervene in several environmental problems caused by SRB, such as corrosion of steel, concrete, and electrode (20); souring of oil (21); altering mobility of toxic heavy metals (e.g., Cr and U) (22); and providing for syntrophic growth with other microorganisms (e.g., methanogens) (23). *Desulfovibrio vulgaris* Hildenborough (*Dv*H), a model SRB strain, was reported to cause the corrosion of carbon steels due to their ability to harvest extracellular electrons from elemental iron oxidation (24). The intracellular electron transport of *Dv*H from the electron donor (i.e., lactate) to the electron acceptor (i.e., sulfate) was proposed via two ways: (i) hydrogen cycling pathway that uses hydrogen as an intermediate electron carrier between the periplasm and the cytoplasm, and (ii) a pathway that bypasses hydrogen cycling and transfers electrons directly to the membrane-bound menaquinone pool (25). However, there is little in-depth knowledge regarding the EET including direct electron transfer and indirect electron transfer from *Dv*H to electrodes. It was found that *D. ferrophilus* IS5 was able to adopt the multi-heme cytochromes containing at least four heme-binding motifs in acquiring energy from solid electron donors (26). Kang et al. found that *D. desulfuricans* was able to conduct direct electron transfer through cytochrome *c* proteins (27). However, so far, no outer membrane *c*-type cytochromes of *Dv*H have been identified (28). Deng et al. (29) found that *Dv*H biosynthesized iron sulfide (FeS) nanoparticles on the cell membrane, which could enhance extracellular electron uptake significantly. Zhou et al. (30) also demonstrated the accumulation of iron sulfide crystallite on the surface of the cell by obtaining electrons intracellularly. Thus, one of the direct electron transfer mechanisms of *Dv*H could be via iron sulfide nanoparticles. Moreover, *D. desulfuricans* utilized electrically conductive nanoscale filaments to transfer electrons to insoluble electron acceptors (i.e., iron(III) oxide) (31). However, the major characteristics of these filaments of *D. desulfuricans* have not been identified. *Dv*H could also form a biofilm, which is dependent on protein filaments such as flagella and pili (32). However, the role of pili, flagella, and relevant biofilm in *Dv*H EET has not been investigated. Flavins, such as riboflavin and flavin adenine dinucleotide (FAD), are well-known electron shuttles (33), which carry electrons among multiple redox reactions and play an important role in indirect electron transfer. An investigation of the EET mechanisms both to and from *Dv*H to the electrode will not only fill the knowledge gap but also encourage the extensive utilization of MFCs in the treatment of sulfate-containing wastewater by SRBs.

**Fig 1:**
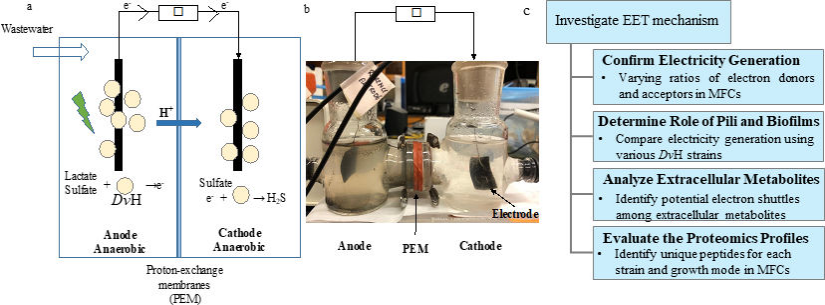
Schematic (**a**) and laboratory-scale prototype (**b**) of the MFC. A schematic representation of experimental flow (c).

In this study, we investigated the different EET mechanisms employed by SRB when growing with the anode as the electron acceptor and the cathode as the electron donor in an anaerobic MFC system. First, this study aimed to determine the effects of the electron donor (i.e., lactate)/electron acceptor (i.e., sulfate) ratio in the anodic chamber and cathodic chamber, respectively, on the electricity generation (Fig. 1c). This investigation can confirm the capacity of *Dv*H in the electricity generation in MFC using lactate and sulfate. To further unveil the role of pili and biofilm in EET of *Dv*H, the electricity generation of MFCs inoculated with *Dv*H JWT700, *Dv*H JW3422 (a mutant with a deletion of the gene coding for the pilin protein), and *Dv*H JWT716 (a mutant has a deficiency in biofilm formation) (34) was compared separately. Subsequently, extracellular metabolites of *Dv*H JWT700, *Dv*H JW3422, and *Dv*H JWT716 under electroactive respiration were analyzed to screen for potential electron shuttles. Finally, the conductivity of the surface structures of *Dv*H and their role in electricity generation were evaluated. Although having *Dv*H in both chambers of the MFC is neither typical nor what would be utilized in an industrial setting, we confirmed that *Dv*H could employ different EET mechanisms depending on its growth mode. These findings will allow for future improved design and implementation of SRB on MFCs or to treat sulfate-containing wastewaters.

## MATERIALS AND METHODS

### Bacterial strains and culture cultivation

Three strains of *Desulfovibrio vulgaris* Hildenborough were used in this study: JWT700 (wild-type, biofilm-forming), JWT716 (mutant of JWT700, non-biofilm forming), and JW3422 (mutant of JWT700, lacks pilin). All *Dv*H strains were isogenic of wild-type, biofilm-forming strain JWT700. Strain *Dv*H JWT716 is a laboratory-driven evolution-derived mutant of JWT700 from a previous study. A single nucleotide polymorphism in the type I secretion system ABC transporter gene blocks the export function of the protein, resulting in a deficiency in biofilm formation due to the lack of excreted polymers for biofilm formation and attachment (34). The third strain is *Dv*H JW3422, which is a mutant containing the deletion of the gene coding for the pilin protein, pilA, or DVU2116. This pilA mutant strain was constructed using the same marker replacement plasmid and method that was previously used to generate *Dv*H strain JW9003 in another study (35). Briefly, the plasmid for deleting DVU2116 and replacing it with a kanamycin resistance gene was transformed into *Dv*H JWT700, and deletion was confirmed by Southern Blot, yielding *Dv*H strain JW3422.

*Dv*H strains were grown anaerobically for 15 h at 34°C on lactate (60 mM), sulfate (30 mM), and a nutrient medium, named MOYLS4 (MgCl_2_, 8 mM; NH_4_Cl, 20 mM; CaCl_2_, 0.6 mM; NaH_2_PO_4_·H_2_O, 2 mM; FeCl_2_, 0.06 mM; EDTA, 0.12 mM; Thioglycolate, 1.2 mM; 6 mL of trace elements solution per liter; and 1 mL of Thauer’s vitamin solution per liter) with the pH adjusted to 7.2. The trace elements solution contained 0.5 g/L MnCl_2_, 0.3 g/L CoCl_2_, 0.2 g/L ZnCl_2_, 0.05 g/L Na_2_MoO_4_, 0.02 g/L H_3_BO_3_, 0.1 g/L NiSO_4_, 0.002 g/L CuCl_2_, 0.006 g/L Na_2_SeO_3_, and 0.008 g/L Na_2_WO_4_. Thauer’s vitamin solution contained 2 mg of biotin, 2  mg of folic acid, 10  mg of pyridoxine HCl, 5  mg of thiamine HCl, 5  mg of riboflavin, 5  mg of nicotinic acid, 5  mg of D calcium pantothenate, 0.1  mg of vitamin B12, 5  mg of *P*‐aminobenzoic acid, and 5  mg of lipoic acid in 1 L of deionized water (36). These media were flushed with nitrogen gas for 15 min in Balch anaerobic tubes sealed with a butyl rubber stopper before use. In this medium, the metabolism of *Dv*H results in the production of H_2_S, which reacts with Fe and generates FeS (black color). The anaerobic tubes were inoculated with a log phase culture of *Dv*H to an optical density of 0.8 at 600 nm (OD_600_). The bacterial absorbance was measured at 600  nm using a spectrophotometer (GENESYS 10S UV‐VIS, Thermo Scientific, USA).

### MFC construction and operation

Double-chambered MFCs were fabricated with a working volume of approximately 90 mL for each compartment and were used throughout the study (Fig. 1). Both anode and cathode were made of carbon cloths (60 wt% Vulcan XC-72 and loaded with Pt 0.5 mg/cm^2^, Fuel Cell Store, USA). Nafion N117 (Fuel Cell Store, USA) was used as a proton exchange membrane (PEM) in the system and pretreated as it was described in the literature (37). The electrodes were connected using platinum wire. The distance between the anode and the cathode was approximately 10 cm. The MFC was operated under a constant external resistance of 1 kΩ using a pure culture of *Dv*H cells growing in both the anodic and cathodic chambers. The bottles and accessories were autoclaved or sterilized before they were assembled.

The anodic chamber and cathodic chamber of the MFC were inoculated with modified MOYLS4 media that contained different concentrations of lactate and sulfate as shown in Table 1. The anodic chamber was fed with 60 mM lactate, whereas 0 mM lactate was provided in the cathodic chamber. Both anodic and cathodic chambers were operated with an initial pH of 7.2 in all experimental runs. All modified MOYLS4 media for each run were autoclaved at 121°C for 20 min. The anode and cathode were kept anaerobic by sparging filter-sterilized nitrogen gas for 15 min at the beginning of each run. In each run, *Dv*H seed culture with an optical density (OD_600_) of 0.8 was used to inoculate each anodic and cathodic chamber to achieve an initial cell concentration of approximately 10^7^ cells/mL right after inoculation. Agitation was maintained at 50 rpm to minimize mechanical shear forces.

**TABLE 1:** MFC experimental conditions

Experimental run (anode| cathode)	Strain	Size of anode (cm × cm)	Lactate in anode chamber (mM)	Sulfate in anode chamber (mM)	Lactate in cathode chamber (mM)	Sulfate in cathode chamber (mM)
Control (0:0|0:0)	*Dv*H JWT700 (Wild type, biofilm+)	2 × 2	0	0	0	30
Run 1 (60:0|0:10)		2 × 2	60	0	0	10
Run 2 (60:10|0:10)		2 × 2		10		10
Run 3 (60:20|0:10)		2 × 2		20		10
Run 4 (60:30|0:10)		2 × 2		30		10
Run 5 (60:10|0:0)		2 × 2		10		0
Run 6 (60:10|0:20)		2 × 2		10		20
Run 7 (60:10|0:30)		2 × 2		10		30
Run 8 (60:20|0:30)		2 × 2		20		30
Run 9 (60:20|0:30)		3 × 3		20		30
Run 10 (60:20|0:30)	*Dv*H JW3422 (pilin-)	2 × 2		20		30
Run 11 (60:20|0:30)	*Dv*H JWT716 (biofilm-)	2 × 2		20		30

Run 1 (anode lactate to sulfate concentration: 60:0 | cathode lactate to sulfate concentration: 0:10) to run 7 (60:10|0:30) was designed to investigate voltage generation at different sulfate concentrations in the anodic chamber and cathodic chamber, respectively (Table 1). For run 1 to run 7, *Dv*H JWT700 was applied, and the electrode size was 2 cm × 2 cm. A control experiment (0:0|0:0) with 30 mM Na_2_S added in the anodic chamber to investigate the role of S^2-^ in the electron transfer with *Dv*H was conducted. Optimal condition (the lactate and sulfate concentrations in each chamber) for potential maximum voltage production based on the results of run 1 to run 7 was proposed, where the ratio of lactate to sulfate is 60:20 in the anodic chamber and 0:30 in the cathodic chamber. Runs 8 (*Dv*H JWT700; 2 cm × 2 cm), 9 (*Dv*H JWT700; 3 cm × 3 cm), 10 (*Dv*H JW3422; 2 cm × 2 cm), and 11 (JWT716; 2 cm × 2 cm) were operated under this condition (60:20|0:30). To investigate the effect of anode size on voltage generation, run 9 adopted a larger anode size (3 cm × 3 cm) compared with run 8 (2 cm × 2 cm). *Dv*H JW3422 and JWT716 strains were applied with an anode and cathode size of 2 cm × 2 cm to test the voltage production without pili and biofilms in run 10 and run 11, respectively. All experiment runs were incubated at room temperature (25 ± 1°C) throughout the operation. Duplicate experiments were conducted for each run. Voltages across the electrodes were measured over time during MFC operation until the voltages decreased to zero. Samples were obtained at the end of each operation to measure pH, acetate, and sulfate concentration after filtration (0.22 µm nylon syringe filter).

### Chemical analyses and electricity generation calculations

Acetate concentrations were quantified by HPLC (Shimadzu LC-20A) equipped with a Supelcogel C610H (30 cm × 7.8 mm) column (Supelcogel, PA) (38). The mobile phase was 0.1% H_3_PO_4_, and HPLC samples were measured at a wavelength of 210 nm. Sulfate concentrations were determined spectrophotometrically (39). Voltage was carried out in the open circuit potential time mode using a CHI600 (Austin, TX, USA) electrochemical workstation. The current was calculated using Ohm’s law, *I* = *V*/*R*, where *I* = current (mA), *V* = voltage (mV), and *R* = resistance (Ω). The power density (W/m^2^) was normalized by the surface area of the anodic chamber. The coulombic efficiency (C_E_) was calculated using the following equation: *C_E_* = *C*_P_/*C*_T_ × 100%, where *C*_P_ is the harvested coulombs calculated by integrating the current over operation time, and *C*_T_ is the theoretical number of coulombs that can be produced from the substrate used. *C_T_* was calculated using the formula: *C_T_* = *F*nSv, where *F* is Faraday’s constant (96,487 C mol^−1^ electron), n is the mole number of electrons produced per mole of substrate oxidation (*n* = 4), S is the substrate (lactate) concentration (mol), and v is the effective volume of the MFC (L). The energy efficiency was calculated as *η*_E_= *E_p_*/*E_T_* × 100%, where *E_p_* is the harvested energy (in joules) calculated by integrating the power (*P* = I × V) over operational time, and *E_T_* is the theoretical value of available energy, obtained from the change in Gibbs free energy, Δ*G*, of −160.1 kJ mol^−1^ lactate oxidation with sulfate as the electron acceptor (40).

### Electrochemical analysis

Cyclic voltammetry (CV) was employed to study the electrochemical performance of electrodes and media with various *Dv*H mutants. Anodes and cathodes from stable running MFCs (run 8, run 10, and run 11) were thoroughly rinsed with phosphate-buffered saline (pH 7.4, 137 mM NaCl, 2.7 mM KCl, 8 mM Na_2_HPO_4_, and 2 mM KH_2_PO_4_) and assembled as working electrodes in a three-electrode cell with Pt wire as counter electrode, Ag/AgCl (sat. KCl, 222 mV vs. SHE) electrode as the reference electrode, and phosphate-buffered saline as supporting electrolyte. The reference electrode was disinfected with 75% ethanol and was then placed in the vicinity of the working electrode surface during CV measurements. The scan rate was 10 mV/s over a range from −0.8 to +0.6 V vs. Ag/AgCl. Electrochemical impedance spectroscopy (EIS) was performed to elucidate the resistance characteristics for various anodes and cathodes with an AC signal amplitude of 10 mV. Charge transfer resistances were obtained by external equivalent circuit fitting analysis of the EIS data, using Randle’s circuit. For the culture media in anodic and cathodic chambers, 50 mL suspension from each chamber in run 8 (*Dv*H JWT700; 2 cm × 2 cm), 10 (*Dv*H JW3422; 2 cm × 2 cm), and 11 (*Dv*H JWT716; 2 cm × 2 cm) were collected to be tested by CVs with glassy carbon as the working electrode, Ag/AgCl (sat. KCl, 222 mV *vs.* SHE) electrode as the reference electrode, and Pt wire as the counter electrode. The CV scan rate was 10 mV/s ranging from −0.6 V to +0.7 V vs. Ag/AgCl.

### Scanning electron microscopy (SEM)

The electrodes from both the anodic and cathodic chambers were obtained from run 8, run 10, and run 11, respectively. A 5 mm × 5 mm section of carbon cloth was cut off from each electrode for SEM analysis. The fixation procedure was done as described elsewhere (31). Briefly, electrode samples were placed in a fixative that contained 2.5% glutaraldehyde (wt/vol), 2.0% paraformaldehyde (wt/vol), and 0.05 mM sodium cacodylate buffer (pH 7.0). The biofilms were fixed overnight and were then washed four times with double-distilled water. Electrode samples were dehydrated by incubation in increasing concentrations of ethanol and then dried. A FEI Quanta 600 FEG SEM was used to examine the surfaces of the biofilm formation on the electrode surfaces. Along with the SEM, X-ray energy dispersive spectroscopy (EDS) mapping was employed to detect C, O, N, S, F, and Fe elements on the surface of Pt-treated conversion coating.

### Conductivity measurements of the surface structure

A 10 mm × 10 mm piece of carbon cloth was cut off from the anode and cathode of MFC run 8 (60:20|0:30). Electrode samples were pretreated using the same fixation procedure described above. Topography images were measured in ambient conditions using tapping mode atomic force microscopy. The local conductivity was measured using the contact mode of a Veeco Dimension V scanning probe microscope. Morphology characterization was completed in tapping mode using Veeco RTESP or OTESPA probes. For stable current-voltage characterization (CVC) responses, applied voltage bias was 2 V and 4 V, respectively, for each electrode sample. The maximum applied bias of the module was 10 V. The CVC response of the plain carbon cloth was measured as well as the control.

### Protein concentration on electrodes and in suspensions

MFC setups that cultivated with *Dv*H JWT700 (wild-type), *Dv*H JW3422 (pilin-), and *Dv*H JWT716 (biofilm-) (i.e., run 8, run 10, and run 11), respectively, were repeated and operated for 5 days. After 5 days, 10 mL solution samples were obtained from each chamber as the suspension sample for protein concentration measurement and peptide sequence analysis. To measure the total protein concentration on the electrodes, each electrode was immersed in 10 mL lysis buffer, which contains 50 mM Tris-HCl and 200 mM NaCl. Then, all these samples were sonicated on ice for 5 min (with 30-sintervals to prevent overheating between each 1-min cycle) and centrifuged at 8,000 × *g* for 20 min to obtain the supernatants, which were used to measure the protein concentrations. A portion of each of the samples from run 8 (*Dv*H JWT700; 2 cm × 2 cm), 10 (*Dv*H JW3422; 2 cm × 2 cm), and 11 (*Dv*H JWT716; 2 cm × 2 cm) was stored in ‒80°C for peptide sequence analysis later. The total protein concentration was measured using the Bradford protein assay, and the absorbance of samples was measured at 595  nm. Bovine serum albumin (BSA) was used as standard and measured in a concentration range of 5 to 100 µg/mL along with the analysis of each unknown sample.

### Proteomics

#### Enzymatic “in liquid” digestion, TMT labeling, and high pH separation

Proteomics was conducted in triplicate for all samples. *Dv*H cell suspensions (1 mL) were transferred to a 2 mL microcentrifuge tube, and 125 µL of protease inhibitors was added immediately (10 × complete Mini from Roche) along with 125 µL of 10% SDS. Cell lysis was conducted by probe sonication with 2 × 20 s intervals at 3 W setting with cooling on ice in between. Samples were subsequently spun for 4 min at max speed (room temperature) to pellet cellular debris, and the supernatant (1,250 µL) was transferred to a new 2 mL tube. Protein precipitation was initiated next by 200 µL addition of 100% trichloroacetic acid and 550 µL of cold acetone, and samples were incubated on ice for 1 h and then spun for 10 min at max speed (room temperature). Generated protein pellets were washed twice with cold acetone, once with cold 80% methanol then finally with 100% cold methanol and air dried. Pellets were solubilized in 8 M urea in 25 mM NH_4_HCO_3_ (pH 8.5), 5 µL aliquot was taken for bicinchoninic acid protein measurement, and the rest was used for tryptic/LysC digestion where the samples were first reduced with 1 mM dithiothreitol for 15 min at 56°C. After cooling on ice to room temperature, 2.6 mM chloroacetamide was used for alkylation where samples were incubated in darkness at room temperature for 15 min. This reaction was quenched with 3.7 mM dithiothreitol. Subsequently, trypsin/LysC solution [100 ng/µL 1:1 Trypsin (Promega):LysC (FujiFilm) mix in 25 mM NH_4_HCO_3_] was added for ~1:40 enzyme:substrate ratio, and 25 mM NH_4_HCO_3_ (pH 8.5) was added to the samples for a final 100 µL volume. Digests were carried out overnight at 37°C then subsequently terminated by acidification with 2.5% trifluoroacetic acid to 0.3% final. 10% heptafluorobutyric acid was added to 0.2% final, and each individual sample was cleaned up using 100 µL Bond Elut OMIX C18 pipette tips (Agilent) according to the manufacturer’s protocol. Eluates in 70%:30%:0.1% acetonitrile:water:TFA acid (vol:vol) were dried to completion in the speed-vac and reconstituted in 25 µL (Low-abundance), 50 µL (Medium-abundance), or 100 µL (High-abundance) of 100 mM triethylammonium bicarbonate for TMTpro labeling with 5 µL for low abundance, 10 µL for medium abundance, and 20 µL for high abundance of TMTpro reagent (TMTpro 16plex Labeling Reagent Set Lot#XE350091 at 12.5 µg/µL from Thermo Scientific) done at room temperature for 1 h with intermittent gentle vortexing. Digested bovine serum albumin internal protein standard was added during labeling to each sample for downstream normalization (2 µg for high, 1 µg for medium, and 500 ng for low set). Additionally, unused TMT channels per each set were used to label a pooled control sample (even an aliquot of each individual sample) to be used as a possible normalization standard between independent sets. The labeling reaction was terminated with the addition of 5% hydroxylamine (0.2% final) and 15-min incubation at room temperature. Master-pool samples were generated by combining all of the individual labeled reactions of each set, freezing at −80°C, and drying to completion using speed-vac. Subsequently re-solubilized with 0.3% TFA/0.2% HFBA and solid-phase extracted with Pierce C18 spin tips (low-set in 200 µL containing ~68 µg total digested protein) or Phenomenex Strata-X 33 µm polymeric column (10 mg/1 mL size for medium set in 400 µL containing ~380 µg total digested protein or 30 mg/3 mL size for high set in 800 µL containing ~860 µg total digested protein) according to the manufacturer’s protocol. Eluates were dried and finally reconstituted in 0.1% formic acid/3% acetonitrile to 1.4 µg/µL concentration for each set.

#### NanoLC-MS/MS

Peptides were analyzed by Orbitrap Fusion Lumos Tribrid platform, where 2 µL was injected using Dionex UltiMate3000 RSLCnano delivery system (ThermoFisher Scientific) equipped with an EASY-Spray electrospray source (held at constant 50°C). Chromatography of peptides prior to mass spectral analysis was accomplished using a capillary emitter column (PepMap C18, 2 µM, 100 Å, 500 × 0.075 mm, Thermo Fisher Scientific). NanoHPLC system delivered solvents A: 0.1% (vol/vol) formic acid and B: 80% (vol/vol) acetonitrile, 0.1% (vol/vol) formic acid at 0.30 µL/min to load the peptides at 2% (vol/vol) B, followed by quick 2-min gradient to 5% (vol/vol) B and gradual analytical gradient from 5% (vol/vol) B to 62.5% (vol/vol) B over 203 min when it concluded with rapid 10-min ramp to 95% (vol/vol) B for a 9-min flash-out. As peptides eluted from the HPLC-column/electrospray source survey MS scans were acquired in the Orbitrap with a resolution of 60,000 followed by HCD-type MS2 fragmentation into Orbitrap (36% collision energy and 30,000 resolution) with 0.7 m/z isolation window in the quadrupole under ddMSnScan 1 second cycle time mode with peptides detected in the MS1 scan from 400 to 1400 m/z; redundancy was limited by dynamic exclusion and MIPS filter mode ON.

#### Proteomics data analysis

Raw data were directly imported into Proteome Discoverer 2.5.0.400 where protein identifications and quantitative reporting were generated. Seaquest HT search engine platform was used to interrogate Uniprot *Desulfoovibrio vulgaris* reference proteome database (UP000002194, 09/28/2022 download, 3,519 total entries) along with a cRAP common lab contaminant database (116 total entries). Cysteine carbamidomethylation and TMTpro-specific labeling were selected as static modifications, whereas methionine oxidation and asparagine/glutamine deamidation were selected as dynamic modifications. Peptide mass tolerances were set at 10 ppm for MS1 and 0.02 Da for MS2. Peptide and protein identifications were accepted under strict 1% FDR cutoffs with high confidence XCorr thresholds of 1.9 for z = 2 and 2.3 for z = 3. For the total protein quantification processing, reporter ion quantifier settings were used on unique and razor peptides, and protein grouping was considered for uniqueness. Reporter abundance was based on BSA-normalized peptide amount intensity values, scaled on all averages and with the co-isolation threshold filter set at ≤40. ANOVA (individual proteins) hypothesis was executed without imputation mode being executed.

#### Untargeted metabolomics using high-resolution LC-MS

Individual 20 mL suspension samples from anode and cathode chambers were obtained and analyzed separately from run 8, run 10, and run 11, respectively. Samples were centrifuged at 4,000  rpm for 10 min, and the supernatant was transferred to a new tube for metabolite extraction. Five milliliters of the supernatants were mixed with the same volume of a 2:2:1 acetonitrile-methanol-water mixture and shaken vigorously. Samples were then placed at −20°C overnight to allow any proteins and cell debris to precipitate. Then, the samples were centrifuged for 15 min at 13,000 rpm and 4°C. Supernatants were transferred to new tubes and dried in a Thermo SpeedVac at 10°C. Dried metabolite extracts were reconstituted in volumes of acetonitrile: water (1:1, vol/vol) normalized to protein content in the sample as determined using NanoDrop Protein A280 measurement mode.

Mass spectrometry data were acquired by running a Thermo Scientific Q Exactive HF Orbitrap LC-MS/MS system in both positive and negative ion modes. Accucore Vanquish C18 column (2.1 × 100 mm, 1.5 µm, Thermo Scientific) and Accucore 150-Amide-HILIC (4.6 × 100 mm, 2.6 µm, Thermo Scientific) were used in the separation of metabolites in positive and negative modes, respectively, with a 5 µL injection volume. Full MS-ddMS^2^ detection mode was applied. For LC, the mobile phases comprised water containing 0.1% formic acid (A) and acetonitrile containing 0.1% formic acid (B). For the reverse phase analysis, metabolites were separated by gradient elution at a flow rate of 0.25 mL/min starting at 5% (vol/vol) B, held for 5 min, increased to 99.5% B within 20 min, and reverted to 5% B at the 30th min, held for 2 min, with a total run time of 32 min. For the HILIC analysis, metabolites were separated by gradient elution at a flow rate of 0.5 mL/min starting at 1% (vol/vol) A, increased to 35% A within 15 min, then to 60% A at the 18th min, and reverted to 1% A at the 20th min, with a total run time of 20 min.

The mass spectrometer was operated as follows: spray voltage 3.5 kV and capillary temperature 350°C. The flow rates of sheath gas, aux gas, and sweep gas were set to 50, 2, and 0, respectively. Full MS resolution was set to 120,000, full MS AGC target was 3 × 10^−6^ with a maximum IT of 250  ms. The scan range was set to 100–1,000 m/z. For MS2 spectra, the AGC target value was set to 2 × 10^−5^, isolation width was set to 1  m/z. The resolution was set to 15,000 and the normalized collisional energy was 40. The dynamic exclusion duration was set to 1.5 s.

Raw data files were converted and then processed using XCMS Online (41, 42) as a multigroup job comparing under different electroactive respiration conditions. For each sample, the filtered features data table was annotated via an accurate mass search against METLIN (43). A targeted analysis of the data was then carried out to determine the behavior of known electron transfer molecules.

## RESULTS

### Electricity generation in the MFCs under different sulfate concentrations

**Fig 2:**
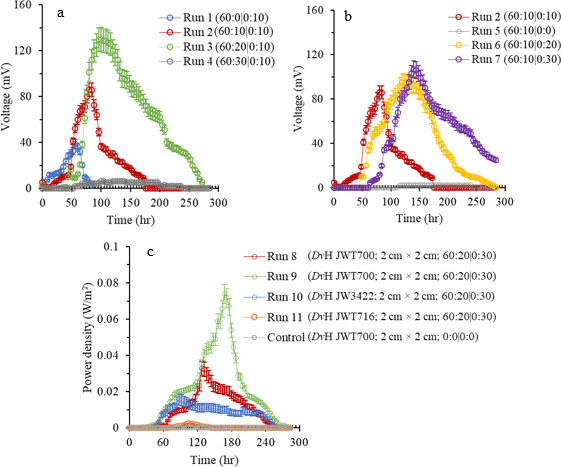
Effects of lactate/sulfate ratio to the anode chamber (**a**) and cathode chamber (**b**) on electricity generation. (**c**) Effect of electrode size and different *Dv*H stains in the anode chamber on powder density. Runs 1–9 and control: DvH JWT700 strain (biofilm+), 2 × 2 cm anode with 60 mM lactate, cathode with 0 mM lactate. Runs 1–4: Anode sulfate varied (Run 1: 0 mM; Run 2: 10 mM; Run 3: 20 mM; Run 4: 30 mM) with cathode at 10 mM sulfate. Control: Anode 0 mM sulfate, cathode 0 mM sulfate. Runs 5–7: Anode 10 mM sulfate, cathode sulfate varied (Run 5: 0 mM; Run 6: 20 mM; Run 7: 30 mM). Runs 8, 10 (DvH JW3422, pilin-), 11 (DvH JWT716, biofilm-): Anode 20 mM sulfate, cathode 30 mM sulfate. Run 9: 3 × 3 cm anode, same conditions as Run 8.

To examine the feasibility and optimal conditions for electricity generation by using *Dv*H JWT700 in the MFC, different sulfate (electron acceptor) concentrations were tested in two chambers, where a fixed concentration of lactate (electron donor; 60 mM) was applied to the anodic chamber, whereas no lactate was provided in the cathodic chamber. The half-reaction on the anode is listed as follows: C_3_H_5_O_3_^-^ + H_2_O → C_2_H_3_O_2_^-^+4e^-^+4H^+^+CO_2_. On the cathode, the reduction of sulfate occurs as follows: SO_4_^2-^+ 10H^+^+ 8e^-^→ H_2_S + 4H_2_O. According to the stoichiometry coefficient, run 1 (60:0|0:10), run 2 (60:10|0:10), run 3 (60:20|0:10), run 4 (60:30|0:10), run 5 (60:10|0:0), and run 6 (60:10|0:20) can theoretically utilize 33.3%, 66.7%, 100%, 100%, 33.3%, 100%, and 100% of the lactate. Among different treatments, electricity generation was observed for run 1 (60:0|0:10), run 2 (60:10|0:10), and run 3 (60:20|0:10), where the maximum electricity generation increased with the increase in sulfate concentration (Fig. 2a). However, as for run 4 (60:30|0:10), no significant electricity generation was observed.

Moreover, when the concentration of sulfate in the cathodic chamber increased and the condition in the anodic chamber stayed the same, a higher maximum electricity generation occurred, and the duration of electricity generation extended (Fig. 2b). A longer lag period was observed for the treatments with a higher maximum electricity generation (Fig. 2a and b). Lee et al. found the MFC fed with only sulfate yielded negligible electricity owing to lacking carbon source in a one-chamber continuous flow MFC at 22°C (8). Among these treatments, a maximum voltage of 131 mV was achieved in run 3 (60:20|0:10) (Table 2). The coulombic efficiencies ranged from 0.79% to 3.13%. The corresponding energy efficiency varied from 0.01% to 0.69%. A slight increase in pH was observed at the end of each test in the cathodic chamber. Thus, the condition with a sulfate concentration of 20 mM and 30 mM in the anodic and cathodic chambers, respectively, was proposed for a higher electricity generation (Table 1) (44).

**TABLE 2:** Comparison of electricity generation, coulombic efficiencies, energy efficiency, and pH in MFCs using *Dv*H JWT700 (wild-type, biofilm+) under different treatments

Treatment (Concentrations of lactate and sulfate in anode and cathode, respectively)	Run 1 (60:0|0:10)	Run 2 (60:10| 0:10)	Run 3 (60:20| 0:10)	Run 4 (60:30| 0:10)	Run 5 (60:10| 0:0)	Run 6 (60:10| 0:20)	Run 7 (60:10| 0:30)
Coulombic efficiency, *η*C (%)	0.79 ± 0.01	1.33 ± 0.03	2.83 ± 0.19	0.14 ± 0.01	0.00 ± 0.0	1.83 ± 0.03	2.20 ± 0.03
Energy efficiency, *η*E (%)	0.01 ± 0.0	0.10 ± 0.02	0.69 ± 0.18	0.00 ± 0.0	0.00 ± 0.0	0.31 ± 0.08	0.37 ± 0.06
Maximum voltage observed (mV)	38 ± 2	88 ± 2	129 ± 1	7 ± 0	3 ± 0	95 ± 4	108 ± 3
pH in anodic chamber	7.2 ± 0.2	7.2 ± 0.1	7.2 ± 0.8	8.2 ± 0.3	8.0 ± 0.6	7.20 ± 0.3	7.3 ± 0.5
pH in cathodic chamber	7.6 ± 0.2	7.5 ± 0.6	7.6 ± 0.5	8.1 ± 0.5	7.5 ± 0.3	7.8 ± 0.4	7.9 ± 0.2

In the anode chamber, *Dv*H JWT700 converted 1 mol of lactate to 1 mol of acetate, producing four electrons. The theoretical electron generation in the anode chamber of run 1 (60:0|0:10), run 2 (60:10|0:10), run 3 (60:20|0:10), and run 4 (60:30|0:10) is 0, 80, 160, and 240 e^-^ eq/L, respectively, according to the initial sulfate concentration in the anodic chamber. Anodic current densities increased as the lactate-to-sulfate ratios decreased (from 60:0 to 60:20). However, when the lactate-to-sulfate ratio reached the theoretical stoichiometric value of 2:1, the current densities dropped to zero. Theoretically, under the conditions of increasing lactate/sulfate ratios, the concentration of acetate produced needs to be twice that of sulfate in the anodic chamber to maintain a balance of electron transfer and stoichiometry between lactate and sulfate. However, these conditions resulted in additional acetate production, except at a lactate/sulfate ratio of 2. In the cathodic chamber, 1 mol of sulfate needs eight electrons to be reduced into sulfide. The electrons required in the cathode chamber of run 1 (60:0|0:10), run 2 (60:10|0:10), run 3 (60:20|0:10), and run 4 (60:30|0:10) are 8 ± 1.7, 25.2 ± 4.2, 31.8 ± 5.6, and 1.1 ± 0.7 e^-^ eq/L, respectively, according to the residual sulfate concentration in cathodic chamber (Fig. S1b). This could be due to the electron transfer from anode to cathode. It is important to note that the electron generation calculated based on acetate concentrations is only reliable when all lactate has been consumed for anabolism or biomass synthesis. Further experiments are needed to determine the flux and fate of lactate carbon in the cell.

Similarly, the theoretical electron generation in the anode chamber of run 2 (60:10|0:10), run 5 (60:10|0:0), run 6 (60:10|0:20), and run 7 (60:10|0:30) is the same, which is 80 e^-^ eq/L based on the initial sulfate concentration in the anodic chamber. However, the actual electron generations were 107.6 ± 6.8, 81.3 ± 2.1, 123.2 ± 3.4, and 147.6 ± 5.6 e^-^ eq/L for runs 2, 5, 6, and 7, respectively (Fig. S1c). As the sulfate concentration increased in the cathodic chamber, more acetate was generated in the anodic chamber. Correspondingly, 25.2 ± 3.6, 0.0 ± 0.0, 34.6 ± 2.8, and 66.3 ± 4.3 e^-^ eq/L were required in the cathode chamber for run 2 (60:10|0:10), run 5 (60:10|0:0), run 6 (60:10|0:20), and run 7 (60:10|0:30), respectively (Fig. S1d). This indicated that most of the electrons generated from the anode were transferred to the cathode for sulfate reduction. The absence of electron transfer in run 5 (60:10|0:0) is due to the sulfate in the anodic chamber capturing all electrons produced by dissimilatory sulfate reduction in DvH, where lactate acts as the electron donor. Additionally, there was no required electron transfer from the anodic chamber to the cathodic chamber as the sulfate concentration in the cathodic chamber was zero.

### Effects of different electrode sizes and *Dv*H mutants on electricity generation

Power densities of MFCs using different anode sizes and *Dv*H strains in MFCs were measured (Fig. 2c). It was observed that after a lag phase of 28 h, the electricity started to be produced and gradually declined after 130 h, mirroring visual observations of biomass growth on electrodes (Fig. S2). We hypothesized that increasing the electrode size, providing a larger surface area for microbes to transfer electrons and attach, would result in an increase in the electricity generation for MFCs. As expected, the anode with a larger surface area (run 9; 3 cm × 3 cm) produced a higher electric potential compared with the anode with a smaller surface area (run 8; *Dv*H JWT700; 2 cm × 2 cm) (Table 3). Run 9 (*Dv*H JWT700; 3 cm × 3 cm) exhibited a maximum power density of ~0.074 W/m^2^, indicating that the larger electrode size may not only offer more significant surface areas for *Dv*H but also enhance its attachment (Table 4). Run 10 applying the *Dv*H JW3422, which could not form pili, had a lower electrical output of ~0.015 W/m^2^ compared with run 8 (~0.040 W/m^2^; *Dv*H JWT700) (Fig. 2c). This suggested that the pili formed by *Dv*H JWT700 may contribute to electricity generation. In addition, there is no significant electricity generation output for *Dv*H JWT716, which has a deficiency in biofilm formation in run 11 (*Dv*H JWT716; 2 cm × 2 cm) (Fig. 2c).

**TABLE 3:** Comparison of power generation, coulombic efficiencies, and pH in MFCs with different electrode sizes and different *Dv*H stains in anode chamber

Treatment	Run 8 (*Dv*H JWT700 (biofilm+); 2 × 2 cm^2^)	Run 9 (*Dv*H JWT700 (biofilm+); 3 × 3 cm^2^)	Run 10 (*Dv*H JW3422 (pilin-); 2 × 2 cm^2^)	Run 11 (*Dv*H JWT716 (biofilm-); 2 × 2 cm^2^)
Coulombic efficiency, *η*C (%)	2.65 ± 0.01	5.65 ± 0.05	2.52 ± 0.06	0.36 ± 0.02
Energy efficiency, *η*E (%)	0.56 ± 0.01	2.12 ± 0.06	0.42 ± 0.02	0.02 ± 0.01
Maximum voltage observed (mV)	125 ± 1	248 ± 2	87 ± 3	42 ± 4
pH in anode chamber	7.2 ± 0.2	7.3 ± 0.1	7.5 ± 0.2	7.2 ± 0.2
pH in cathode chamber	7.6 ± 0.3	7.7 ± 0.2	7.7 ± 0.3	7.4 ± 0.3

**TABLE 4:** Protein concentrations on electrodes and solutions for MFC cultivating different *Dv*H strains

	Anodic chamber		Cathodic chamber	
	Anode	Solution	Cathode	Solution
	Total protein (µg)	Protein concentration (µg/mL)	Total protein (µg)	Protein concentration (µg/mL)
Initial setup	0	3.9 ± 2.3	0	3.9 ± 2.3
DvH JWT700 (biofilm+) (run 8)	262.2 ± 6.2	54.9 ± 0.7	8.5 ± 3.1	12.7 ± 0.9
DvH JW3422 (pilin-) (run 10)	186.3 ± 19.6	56.7 ± 1.2	1.1 ± 0.5	10.7 ± 0.5
DvH JWT716 (biofilm-) (run 11)	27.7 ± 3.1	42.0 ± 3.5	0.1 ± 0.0	5.9 ± 0.6

According to the initial sulfate concentration in the anodic chamber, the theoretical electron generation in the anode chamber of run 8 (*Dv*H JWT700; 2 cm × 2 cm), run 9 (*Dv*H JWT700; 3 cm × 3 cm), run 10 (*Dv*H JW3422; 2 cm × 2 cm), and run 11 (*Dv*H JWT716; 2 cm × 2 cm) are the same, which is 160 e^-^ eq/L. However, run 9 with a larger electrode size had a significantly higher electron generation (238.2 ± 6.3 e^-^ eq/L) than run 8 (224.0 ± 3.1 e^-^ eq/L) (Fig. S1e). As such, more sulfate was reduced in run 9 than in run 8 in the cathodic chamber (Fig. S1f). Additionally, run 10 with non-pili-forming strain *Dv*H JW3422 had an electron generation of 189.6 ± 5.4 e^-^ eq/L in anode chamber, whereas run 10 with non-biofilm-forming strain *Dv*H JWT716 produced 161.0 ± 2.3 e^-^ eq/L. Correspondingly, 2.83 ± 0.08 mM and 0.24 ± 0.01 mM sulfate were reduced by using 22.7 ± 0.7 and 1.9 ± 0.08 e^-^ eq/L transferring from anode to cathode for run 10 (*Dv*H JW3422; 2 cm × 2 cm) and 11 (*Dv*H JWT716; 2 cm × 2 cm), respectively. Table 3 also showed that pH in the cathodic chambers significantly increased after different treatments (*P* < 0.01). By conducting a control experiment using Na_2_S to mimic the S^2-^ produced in the anode chamber, no electricity signal was observed. This indicated that S^2-^ was not the main factor for electricity generation.

The initial protein concentration after seeding in the solution in both chambers of MFCs was 3.9 ± 2.3 µg/mL (Table 4). After ~5 days of operation, the protein concentrations in the anodic chamber increased due to the growth of cells (Fig. S2). It was observed that MFCs with *Dv*H JWT700 and *Dv*H JW3422 had higher protein concentrations than the MFC with *Dv*H JWT716 in the anodic chamber. Additionally, the protein concentrations in the cathodic chamber for MFCs with *Dv*H JWT700 and *Dv*H JW3422 increased from 3.9 ± 2.3 µg/mL to 12.7 ± 0.9 µg/mL and 10.7 ± 0.5 µg/mL, respectively. The anode with *Dv*H JWT700 had the highest amount of the total protein (262.2 ± 6.2 µg), followed by the anode with *Dv*H JW3422 (186.3 ± 19.6 µg) and then the anode with *Dv*H JWT716 (27.7 ± 3.1 µg). A similar trend was found for the protein amount on the cathodes with various *Dv*H strains: *Dv*H JWT700 (8.5 ± 3.1 µg) >*Dv*H JW3422 (1.1 ± 0.5 µg)> *Dv*H JWT716 (0.1 ± 0.0 µg) (Table 4). However, the amounts of protein on the cathode were much lower than the ones on the anode.

### Electrochemical analysis of electrodes and electrolytes with different *Dv*H mutants

**Fig 3:**
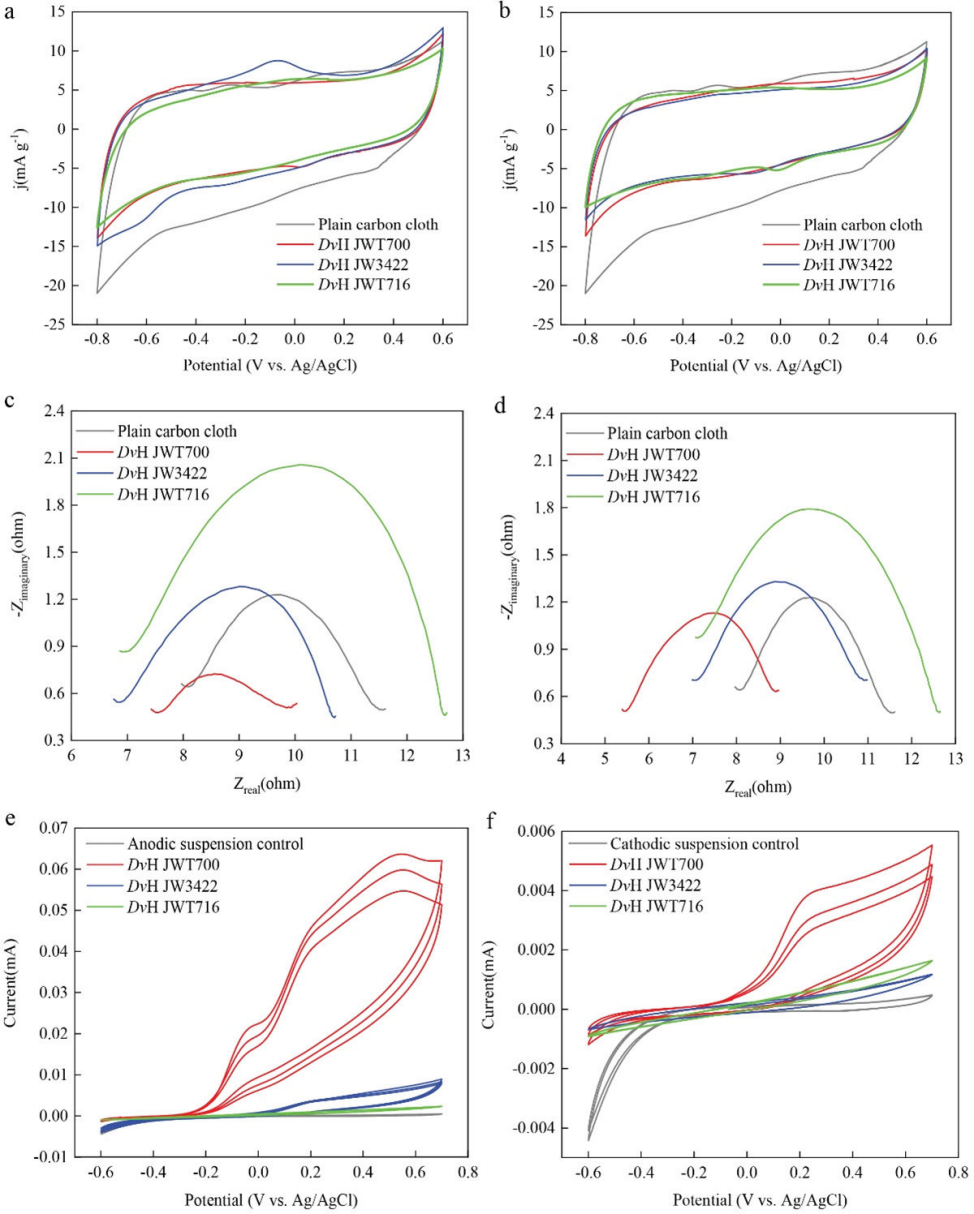
Electrochemical analysis of carbon cloths with different biofilms formed by various *Dv*H mutants. Cyclic voltammograms for the anodes (**a**), cathodes (**b**), anodic media (**e**), and cathodic media (**f**) with different *Dv*H mutants. Nyquist impedance plots of the anodes (**c**) and cathodes (**d**) with different *Dv*H mutants’ biofilms.

CVs were collected using the anodes and cathodes with biofilms formed by *Dv*H JWT700, *Dv*H JW3422, and *Dv*H JWT716, respectively, as working electrodes to determine the effects of *Dv*H mutants on electrodes’ performance (Fig. 3). The plain electrode (i.e., carbon cloths) displayed larger CV curve areas without obvious distortions compared with electrodes with DvH biofilms. This is because biofilms on carbon cloths can cover the electrode surface, increase resistance, and affect electron transfer efficiency. Electrodes with *Dv*H mutants presented a well-defined symmetric shape and similarly enclosed area, suggesting a comparable electric double layer (EDL) capacitive performance. In particular, anodes with *Dv*H JW3422 (lacking pilin) had a remarkable oxidation peak at about −0.05 V vs. Ag/AgCl, although the redox reactions presented the irreversibility without the presence of a mirrored reduction peak. A similar trend was not observed for the cathode with *Dv*H JW3422.

The Nyquist plots analyzed by the classical equivalent electrical circuit are shown in Fig. 3c and d. The diameter of the semicircle represents the charge transfer resistance (R_ct_). The R_ct_ values of anodes with *Dv*H JWT700, *Dv*H JW3422, and *Dv*H JWT716 were found to be around 2.2, 3.7, and 5.7 Ω, respectively. This suggests that the biofilm formed by *Dv*H JW3422 (lacking pilin) may be less conductive than that formed by *Dv*H JWT700 (wild-type). However, drawing a definitive conclusion would require further accumulation of more biofilms on electrodes for a more comprehensive analysis. Since anodes with DvH JWT700 biofilm had lower R_ct_ values than the plain carbon cloths (3.4 Ω), indicating that *Dv*H JWT700 formed the effective biofilm on the anode and promoted the electron transfer of electrodes. However, the cathodes with *Dv*H JWT700 and *Dv*H JW3422 biofilms had similar R_ct_ values with plain carbon cloths.

To investigate the components secreted by *Dv*H strains, further CV analysis was conducted for the anodic and cathodic suspension solutions. As a result, no redox peaks were found when a MOYLS4 medium without bacteria was used as the anolyte and catholyte (Fig. 3e and f). Anodic suspensions with *Dv*H JWT700 and *Dv*H JW3422 in MOYLS4 had an oxidation peak in the forward scan of CVs at 0.45 V vs. Ag/AgCl. During the reverse scan, no reduction peak was present, which suggested oxidation activity because of the important contributions from *Dv*H JWT700 and *Dv*H JW3422. When the oxidation peaks of anodic suspension with *Dv*H JWT700 (wild-type) were much higher than that of *Dv*H JW3422 (lacking pilin), indicating that there were more oxidative components in the anodic suspension secreted by *Dv*H JWT700 than *Dv*H JW3422. Similarly, weaker peaks were exhibited for a cathodic suspension of *Dv*H JW3422 and *Dv*H JWT716 (lacking biofilm) compared with those of *Dv*H JWT700. It indicated that *Dv*H JWT700 secreted more oxidative components in the cathodic chamber. The weaker peaks redox by the cathodic suspension of *Dv*H JWT716 suggested that there might be redox components in the cathodic suspension that were secreted by the *Dv*H JWT716.

### SEM and EDS analysis of different *Dv*H strains on electrode

**Fig 4:**
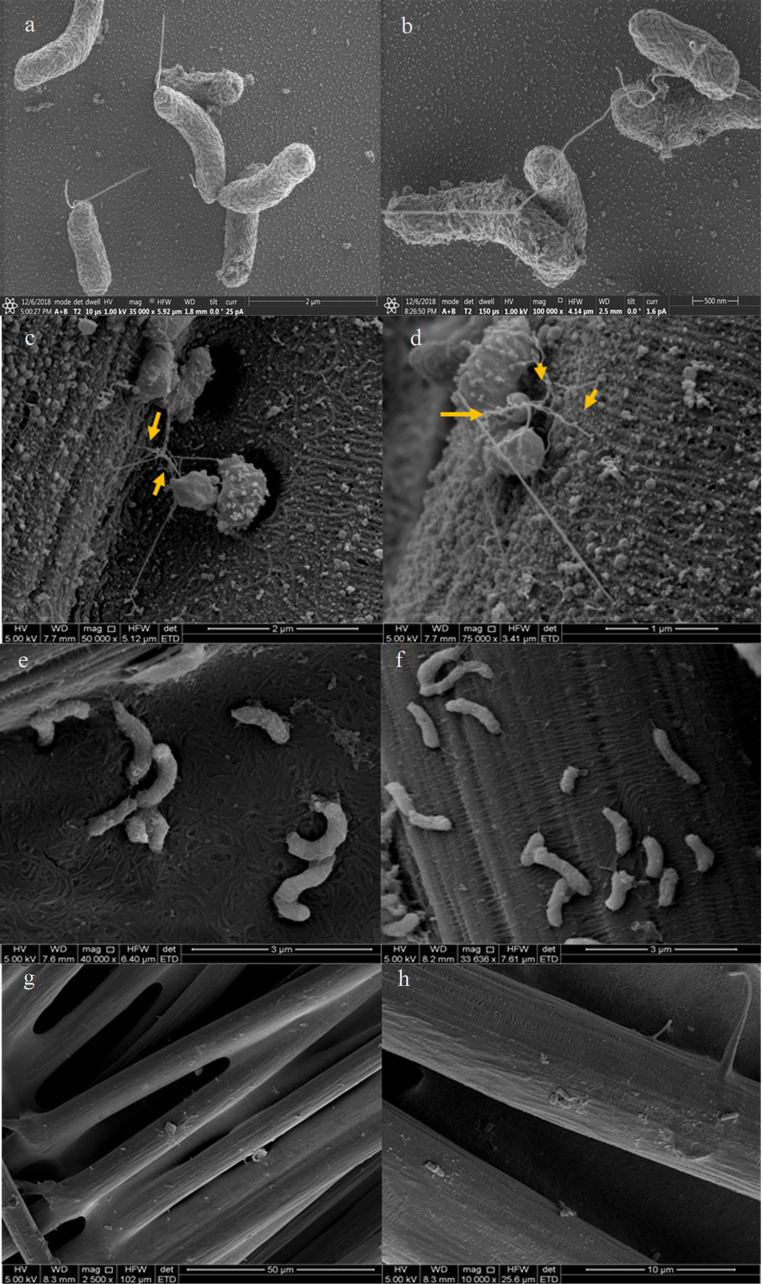
SEM micrographs of *Dv*H JWT700 (biofilm+) (a and b) growing on mica sheets, *Dv*H JWT700 (c and d), *Dv*H JW3422 (pilin-) (e and f) and *Dv*H JWT716 (biofilm-) (g and h) growing on carbon cloths. The yellow arrow represents the filaments formed by wild *Dv*H.

By SEM, we observed that cells are curved rod-shaped (width of ~0.5  µm and length of ~3  µm) (Fig. 4). When growing on a mica sheet, *Dv*H primarily relies on its flagella to attach to the mica sheet surface (Fig. 4a and b). SEM images clearly revealed that biofilm formed by *Dv*H on the electrode surface, which has been retrieved from MFCs. It is worth noting that *Dv*H biofilm formed on the surface of the anode contained a minimal amount of extracellular polymeric substance (EPS) materials that surrounded the cell (Fig. 4c and d; Fig. S4a). In addition, *Dv*H also has pili with lengths of around 2 µm firmly attached to the cathode carbon cloth. The attachment was observed at the end of filaments radiating out from a *Dv*H cell and sometimes between the attachment locations of adjacent filaments from *Dv*H cells. For comparison, *Dv*H JW3422 (lacking pilin) was found to contain EPS but without pili forming on the surface of the electrode (Fig. 4e and f; Fig. S4b). Although most *Dv*H JW3422 cells did not form a dense biofilm, the EDS and EDX indicate attachment to the surface of the cathode which could potentially either be mediated by direct attachment or via EPS networks. As for the MFC cultured with *Dv*H JWT716 (lacking biofilm), there were few cells attaching to the cathode (Fig. 4g and h; Fig. S4c). EDS elemental spectra showed enrichment of C, O, and S in the apparent biomass deposits for *Dv*H JWT700 and *Dv*H JW3422, indicating the presence of biological material and evidence of dissimilatory sulfate reduction compared with electrode-only areas (Fig. S4 d and e).

### cAFM scanning of electrodes with *Dv*H biofilm

The local structure of electrodes with *Dv*H biofilms and electronic properties were investigated through cAFM (Fig. S3). Topographic maps of the electrodes (carbon cloths) with biofilms were conducted by 2 µm × 2 µm to narrow down the location of microbe clusters and pili. The current was detected using a current-to-voltage preamplifier in the center topographic maps. Different voltages (i.e., 0–2 V and 0–4 V, respectively) were applied to plain carbon cloth and the carbon cloth with *Dv*H biofilm. The current signal of the carbon cloth with *Dv*H biofilm fluctuated greatly compared with the plain carbon cloth, which had relatively flat signals (Fig. S3b, d, f, and h). Despite this, the conductivity of *Dv*H biofilm or pili cannot be determined since carbon clothes are highly conductive.

### Proteomics and metabolomics of electroactive respiration in *Dv*H

**Fig 5:**
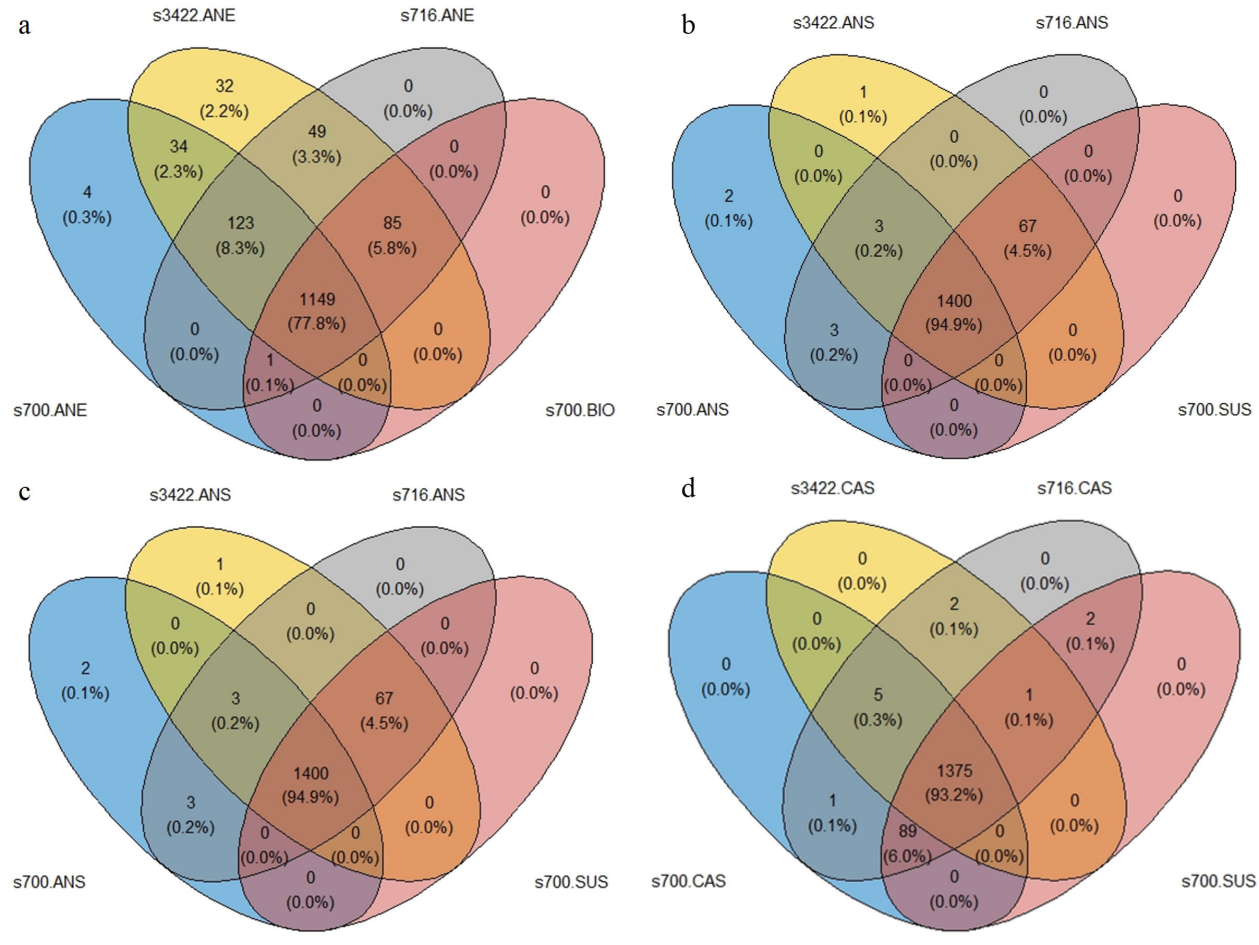
Venn diagram showing the number of unique peptides identified from wild-type, biofilm-forming strain *Dv*H JWT700 (s700; represented by the blue circle), pilin deletion mutant strain *Dv*H JW3422 (s3422; represented by the yellow circle) and biofilm lacking strain *Dv*H JWT716 (s716; represented by the grey circle) across various electroactive respiration modes. The labels ANE and ANS correspond to the anode and the suspension culture in the anodic chamber, respectively, whereas CAE and CAS represent the cathode and the suspension culture in the cathodic chamber, respectively. Additionally, s700.BIO and s700.SUS denote the biofilm and planktonic cultures of DvH JWT700 cultivated without electroactive repatriation modes.

For each sample analyzed by proteomics in triplicate, more than 1,200 peptides were detected (Fig. S5). From a multi-group comparison, a total of 1,149, 1,400, 1,208, and 1,375 peptides were identified and found to be shared among different *Dv*H strains in four different electroactive respiration modes: on the anode (Fig. 5a; Table S1), in the anodic suspension (Fig. 5b; Table S2), on the cathode (Fig. 5c; Table S3), and in the cathodic suspension (Fig. 5d; Table S4), respectively. Upon comparing these peptides with the ones for biofilm and planktonic cultures of DvH JWT700 cultivated without electroactive respiration modes, there is a higher percentage of unique peptides on the anode (comprising 16.4% of the total peptides found on the anode) and the cathode (accounting for 12.2% of the total peptides found on the cathode) relative to biofilm growth using dissimilatory sulfate reduction when compared with their respective suspensions (constituting only 0.6% in the anodic suspension and 0.5% in the cathodic suspension) relative to the spent medium. This suggests significant internal re-wiring in *Dv*H to adapt to electroactive respiration compared with the dissimilatory sulfate reduction respiration of *Dv*H.

When growing in MFCs (electroactive growth mode), an increased presence of pili-related proteins (DVU2118, DVU1262, and DVU0451) and flagella-related proteins (DVU0513, DVU0311, DVU0514, and DVU0739) was noted for *Dv*H JWT700 and *Dv*H JW3422 on the anode (Table S1). These proteins could potentially help cell attachment, biofilm formation, and electron transfer. The unique peptides in the anode for *Dv*H JWT700 (wild-type with biofilm formations) are DVU2118, DVU2092, and DVU2413. DVU2092 and DVU2413 are both uncharacterized proteins. DVU2092 may be related to moeB thiF family protein, contributing to thiamin generation and electron transport chain. DVU2413 is related to radical SAM domain protein, which binds with Fe-S cluster and initiates a range of radical transformations. For *Dv*H JW3422 (pilin-), there are more unique peptides compared with *Dv*H JWT700, including uncharacterized proteins (DVU0401, DVU0975, DVU1359, etc.), proteins related to metabolism and biosynthesis (cytochrome c family protein, glycosyl hydrolase, glycosyl transferase, etc.), proteins involved in signal transduction and regulation (sensor/response regulator, GAF domain/sensory box/EAL domain protein, and TRASH domain-containing protein), and proteins associated with transport systems (AcrB/AcrD/AcrF family protein, CBS/transporter associated domain protein and Peripla_BP_6 domain-containing protein).

On the cathode, a substantial presence of pili- (DVU2227, DVU1273, and DVUA0113) and flagella-related (DVU0519, DVU0910, DVU3227, and DVU0045) proteins was observed for *Dv*H JWT700 and *Dv*H JW3422 (Table S3). These proteins include a histidine kinase, which suggests an increased demand for surface attachment or motility. Furthermore, dominant unique peptides in the cathode for *Dv*H JWT700 (wild-type) and *Dv*H JW3422 (lacking pilin) include periplasmic [NiFe] hydrogenase (DVU2525) and [Fe] hydrogenase (DVU1771), underscoring their significance in the electroactive respiration process.

Fewer unique peptides were observed in the suspension of both the cathode and anode compared with those on the electrodes. Two unique peptides for *Dv*H JWT700 (wild-type) in the suspension of the anodic chamber include DNA-binding response regulator and Ech hydrogenase. The DNA-binding response regulator helps the cell respond to various environmental stimuli, whereas Ech hydrogenase is involved in energy conservation and is part of electron transport chains. These may indicate the adaption of *Dv*H JWT700 (wild-type) in electronic growth mode.

For metabolites that were observed, identified, and validated in both positive and negative modes, we found indications that riboflavin, flavin mononucleotide (FMN), and reduced flavin mononucleotide (FMNH_2_) could be dysregulated between electroactive respiration modes (Table 5; Fig. S8). FMN and FMNH_2_ showed higher intensity in *Dv*H JWT700 (wild-type) and *Dv*H JW3422 (pilin-) in the anodic chamber compared with *Dv*H JWT716 (biofim-), whereas riboflavin showed higher intensity in *Dv*H JWT716 the anodic chamber. By comparing the intensity of riboflavin detected in each sample to the initial concentration of riboflavin (five ppm) in the medium, we noticed that the riboflavin had been utilized at different levels. Riboflavin had potentially been consumed in the anodic chamber when culturing *Dv*H JW3422, which lacks pili. FMN was observed in electroactive respiration modes of *Dv*H mutants. However, FMNH_2_ was produced by *Dv*H JWT700 and *Dv*H JW3422 under electroactive respiration, but not in the cathodic chamber culturing *Dv*H JWT716, which cannot form biofilm. These possible patterns in flavin molecules did correspond with the flavin synthesis peptides observed.

**TABLE 5:** Flavin-related metabolites of *Dv*H strains under different electroactive respiration modes

	RT_med_^*a*^	M/z	Intensity (TIC)					
			DvH JWT700 (Run 8) (wild-type, biofilm+)		*Dv*H JW3422 (Run 10) (pilin-)		*Dv*H JWT716 (Run 11) (biofilm-)	
			An_ suspension	Ca_ suspension	An_ suspension	Ca_suspension	An_ suspension	Ca_suspension
Riboflavin	12.95	357.1197	37,127	113,997	0	90,778	106,478	63,719
FMN	15.16	240.0513	124,739	67,377	133,909	11,407	32,134	20,675
FMNH_2_	9.81	442.102	181,281	18,850	83,510	49,590	32,948	0

^*a*^ The median retention time of peaks in the group.

## DISCUSSION

**Fig 6:**
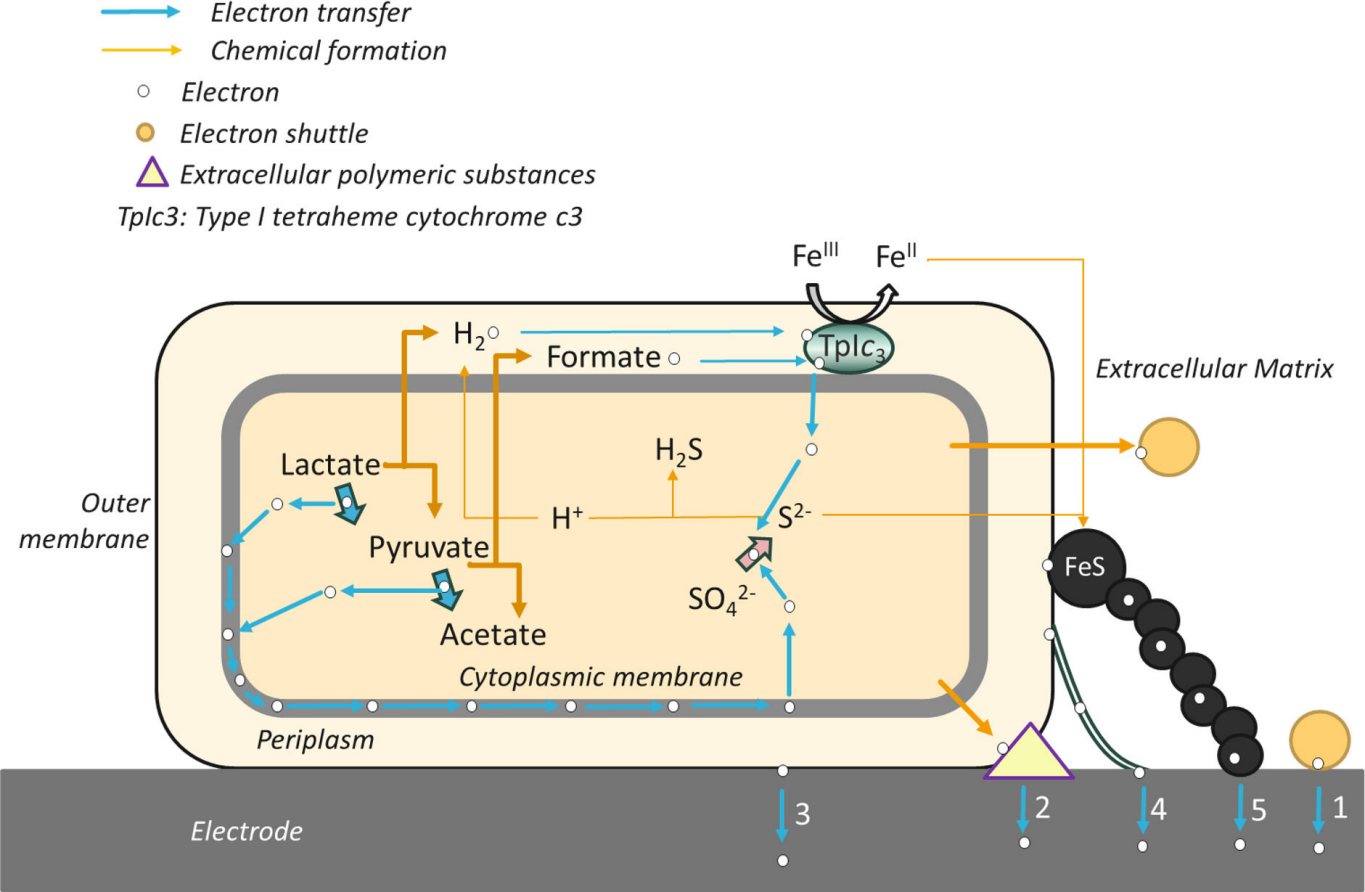
All potential EET mechanisms of *Dv*H. Indirect electron transfer based on electron shuttles (1) and direct electron transfer based on: EPS (2), unknown outer membrane *c*-type cytochromes (3), conductive pili/filaments (4), and FeS clusters (5) are presented.

In this study, we observed that *Dv*H can harvest and send electrons to and from an electrode by varying the concentrations of the electron donor/electron acceptor ratio in the anodic chamber and cathodic chamber, respectively. By testing different *Dv*H mutants (i.e., *Dv*H JW3422 [pilin-] and *Dv*H JWT716 [biofilm-]), we determined that pili and biofilm contributed to the EET processes of *Dv*H. We also identified the extracellular metabolites excreted during growth under electroactive respiration and found molecules that could act as electron shuttles. Our results in conjunction with previous literature for DvH and other members of the *Desulfovibrio* genus led us to draw the schematic in Fig. 6, which shows all the potential mechanisms of EET in DvH that we will now discuss.

It is important to note that these EET mechanisms are distinct from the biocorrosion processes, which are well described for *Dv*H, as corrosion occurs primarily during growth under dissimilatory sulfate-reducing conditions (45). Previous studies focused mainly on the biocorrosive capacity of *Dv*H (46, 47) and overlooked the electricity production capacity of *Dv*H through MFCs. The reported coulombic efficiency in the current study is much lower (Tables 2 and 3) than the ones reported in other relevant studies, which varied from 6.7% to 98.9% when different feed compositions and SRB were applied (8, 48, 49). The highest current densities come from mixed cultures that are usually dominated by the genus *Geobacter* (50). The differences in coulombic efficiency were caused by differences in MFC configurations and SRB species, which have different electron transfer mechanisms or use different electron donors (16). Interestingly, *Dv*H grew on both the anode and the cathode of the MFC while producing electricity, which means that *Dv*H is capable of bidirectional EET. Likewise, the proteomics results showed a complete proteome rearrangement for electroactive respiration in a biofilm compared with DSR biofilm and indicated that different sets of proteins were involved in the use of the anode as the electron acceptor and use of the cathode as the electron donor. From this observation, we then asked how EET was being carried out by *Dv*H and if it involved different EET mechanisms when the anode was used as the electron acceptor and the cathode as the electron donor.

### Possible indirect electron transfer mechanisms: electron shuttle molecules

The first possible mechanism depicted in Fig. 6.1 is indirect electron transfer. In our study of *Dv*H, there were several experimental results that suggested the possibility of indirect electron transfer occurring in this anaerobic bacterium. In the MFC runs with *Dv*H wild-type and mutant strains, it was seen that neither the lack of biofilm nor the lack of pili completely diminished EET. This suggested the involvement of IET. For other bacteria with known IET mechanisms, small molecules called electron shuttles ferry electrons between the cell and the electron source or sink have been characterized. *Shewanella* species were found to secrete flavins (i.e., FMN and riboflavin) as electron shuttles, which mediate EET by binding to outer membrane cytochromes (51). *S. oneidensis* MR-1 can use the interaction of flavin/outer membrane *c*-type cytochrome complexes to regulate extracellular electron transport (52). Although *Geobacter* species have abundant *c*-type cytochromes and are thought to transfer electrons by direct contact, flavin synthesis and excretion genes are widely distributed in *Geobacter* species (53). Studies indicated that *Geobacter sulfurreducens* can uptake self-secreted riboflavin as bound cofactors for EET (54). In the metabolite analysis of DvH, it was not surprising that we detected riboflavin since we provided riboflavin as a component of the media (Table 5). However, under electroactive respiration, *Dv*H strains did have different patterns of riboflavin utilization. Given the experimental setup for these metabolomics experiments, we cannot quantify or provide relative quantitation for these changes. However, in the proteomics experiments which were done quantitatively, we saw increases in the abundance of riboflavin synthesis genes (e.g., DVU1199, DVU1200, and DVU1201) in electroactive conditions compared with *Dv*H under dissimilatory sulfate reduction respiration samples that matched the observations of riboflavin usage in the metabolomics. It was previously found both riboflavin and FAD accelerated pitting corrosion and weight loss on the stainless steel caused by *Desulfovibrio vulgaris* biofilm (53). Flavin-like molecules were also found to increase in electron donor-limiting conditions by mass spectrometry in *Desulfovibrio alaskensis* G20 biofilms (33). Thus, flavins may act as electron shuttles of *Dv*H. In *Dv*H, flavin adenine dinucleotide (FAD) in quinone form which is the electron shuttle could accept two electrons and two protons to become hydroquinone form (FADH_2_) (55).

Further evidence for the possibility of IET in *Dv*H was seen in the cyclic voltammogram, which gave indications of both the effect of some corrosion from hydrogen sulfide as well as the possibility of electron shuttling. CVs indicated the larger EDL capacitance of plain carbon cloths than the electrodes with *Dv*H biofilms (Fig. 3a and b), which can be due to hydrogen sulfide produced by *Dv*H mutants poisoning the Pt wires on the surface of carbon cloths as shown in the schematic in Fig. 1 (56). Those poisonings largely reduced the pseudocapacitive contributions from Pt wires. Furthermore, the lower R_ct_ of the anode with *Dv*H JWT700 (wild-type) demonstrated the formation of more effective biofilm on the anode that enhanced the electron transfer process (Fig. 3d and c) as reported for *Klebsiella variicola* (57). However, non-pili-forming *Dv*H JW3422 and non-biofilm-forming *Dv*H JWT716 did not enhance the electron transfer process of anodes and non-biofilm-forming *Dv*H JWT716 presented less conductive biofilm which may be due to the semiconductive EPS and the poisoning of Pt wires via hydrogen sulfide (56, 58). Irreversible oxidation processes appear in both the anodic and cathodic suspensions of *Dv*H JWT700. In both chambers, the suspension of *Dv*H JWT700 (wild-type) had a larger oxidation current density than *Dv*H JW3422 and *Dv*H JWT716, suggesting that *Dv*H JWT700 secreted more stable and oxidative compounds than the other two. The obtained CV curves are very similar to those in a previous study that identified these compounds as quinone or benzene derivatives, which suggests similar molecules may participate in DvH EET (59). However, the cell-surface redox-active proteins as well as further characterization of small molecule contributions need to be investigated to determine the free-flavin-mediated electron-shuttling mechanism of *Dv*H.

### Possible direct electron transfer mechanisms: *Dv*H utilized filaments that facilitated but not necessarily conducted electron transfer from cell-to-cell and to the electrode

Throughout our various running conditions in the MFC, we consistently observed that greater attachment and larger biofilm led to better electricity generation in parameters such as power density and columbic efficiency, which suggests the possibility for direct electron transfer. Furthermore, in these high-performing runs, microscopy images revealed the presence of biogenic extracellular structures appearing to connect the cell to the electrode surface and frequently other cells in the biofilm. To evaluate the possibilities for direct electron transfer mechanisms in DvH EET, we considered known extracellular structures and enzymes on the cell surface or integral outer membrane proteins.

Biofilms of bacteria are often composed of EPS or extracellular polymeric substances (Fig. 6.2). In our study, a thin biofilm of DvH attached to the electrode surface facilitated by a network of extracellular structures was beneficial for the growth of DvH in electroactive conditions. *Dv*H did not produce an extensive exopolysaccharide matrix and its biofilm formation is reported to be dependent upon protein filaments (32). EPS is not known to be conductive, but it was previously demonstrated that extracellular polymeric substances include polysaccharides, proteins, glycoproteins, glycolipids, and humic substances, which these molecules can possess some semiconductive properties (60). Thus, EPS or biofilm proteins could trap conductive molecules which may be contributing to or mediating a direct electron transfer mechanism. It is more likely however that EPS or the biofilm matrix of DvH provides the structural support or decreased distance that enables EET from the cell to an electrode or conductive surface.

The set of extracellular structures we considered are conductive outer membrane proteins such as outer membrane cytochrome *c*’s (OMCC) (Fig. 6.3). In other bacteria, OMCCs are a well-described direct electron transfer mechanism. Different outer membrane *c*-type cytochromes such as MtrC, OmcA, OmcE, and OmcS offer various routes of electron transfer extracellularly for *S. oneidensis* and *G. sulfurreducens*; however, DvH lacks homologs to any of these characterized direct electron transfer proteins (61, 62). Previous studies demonstrated that *Dv*H contains several membrane-bound redox complexes such as Qrc (quinone reducing complex) and Hmc (high molecular weight cytochrome) complex that can accept electrons (63, 64). Qrc complex can accept electrons from the low-redox potential hemes of a type one soluble cytochrome *c_3_* protein, TpI*c*3, whereas Hmc transferred the electrons by transporting H^+^ from cytoplasmic lactate oxidation to the periplasmic cytochrome *c*_3_ network (65). However, none of these proteins were located at the outer membrane. Additional evidence in the CV had a small redox peak in the case of *Dv*H JW3422 (lacking pilin) revealing that it might contribute to the oxidation reaction through unknown outer membrane *c*-type cytochromes. Despite this, some uncharacterized proteins such as DVU1174, DVU0401, DVU1359, DVU0842, and DVU2997 that we observed on electrodes should be further investigated to determine their functions in EET of *Dv*H but are not predicted to be cytochrome containing and computational predictions of their subcellular localization was inconclusive. Based on findings in a previous study that extracellular enzymes such as hydrogenase and formate dehydrogenase could mediate a direct electron uptake for *Methanococcus maripaludis,* we considered if such enzymes could have a role in *Dv*H EET (66). However, *Desulfovibrio* species mainly have cytoplasm-located and periplasmic hydrogenases that contribute to intracellular EET and hydrogen formation (67, 68). More outer membrane redox complexes need to be investigated to reveal the possibility of direct electron transfer mediated by OM proteins in *Dv*H.

Finally, for direct electron transfer mechanisms, we considered the possibility of conductive nanowires made out of protein (Fig. 6.4) or iron sulfide minerals (Fig. 6.5). A previous study reported that the presence of nanowires of *Candidatus Desulfofervidus* HotSeep-1 cell depended on substrates hydrogen, which contributes to the interspecies electron transfer (69). *Deltaproteobacteria* have many different but not always fully described extracellular electron transfer mechanisms including groups like cable bacteria (70, 71), indicating that *Dv*H may have similar electron transfer mechanisms with other bacteria in *Deltaproteobacteria*, such as *Geobacter* sp., as indicated by protein sequence similarity of their pilins (Fig. S7).

Our results from several experiments indicated that the pili appear to play an important role in mediating the extracellular electron transfer processes in DvH, whether by facilitating attachment or possibly through conduction. Although the SEM-preparatory dehydration process may collapse or separate outer-membrane materials and break filaments, it was observed that although *Dv*H exhibited then biofilm formation, it produced numerous filaments or pili on the surface of carbon cloths (Fig. 4). We noticed that pili and/or filaments were effective for the attachment of cells of *Dv*H JWT700 on the surface electrode or functional as the networks between cells (Fig. 4). However, no such attachment using filaments was observed for mutant strain *Dv*H JW3422 (pilin-). In addition to attachment, pili-related proteins (DVU2118, DVU2227, and DVU1262) were present in the anodic chamber in electroactive conditions but were not found in *Dv*H under dissimilatory sulfate reduction respiration (Fig. 5; Table S1). Crucially, in this study, MFC cultivating non-pili-forming *Dv*H JW3422 exhibited a lower power density than the wild-type strain (Fig. 2). However, no definitive answer as to the conductivity of pili from DvH can be obtained based on the current cAFM findings.

Given the inconclusive results of our experiment, we compared our results and DvH proteins with reports in the literature on conductive pili. Type IV pili can be categorized into two subclasses, type IVa pili and type IVb pili, based on the sequence and length of the pilin subunit (72). *Geobacter sulfurreducens, Geobacter bremensis, Desulfuromonas thiophila*, and so on that were reported to have conductive pilin (e-pili) have type IVa structure (73). Although Holmes et al. (73) reported pili in *Desulfovibrio vulgaris* were the long type IVa pilin, according to the findings in the protein sequence alignment (Figs. S6 and S7), the *Dv*H pilin system is closer to the type IVb system. Until now, the pili structure of *Dv*H has not been well studied. Normally, the conductivity depends on the composition of the amino acid chain of the major pili (74). A high density of aromatic amino acids and a lack of substantial aromatic-free gaps along the length of long pilins may be important features of e-pilin (74). Two pilin proteins were found on the genome of *Dv*H: one is major pili, which belong to Flp family type IVb pili (DVU2116) (Fig. S6), and the other, prepilin-type N-terminal cleavage/methylation domain-containing protein, which may be the minor pili (putative PilE) of *Dv*H (Fig. S7). We further compared the major pilin and minor pili with the e-pili reported previously. No similar trend between sequences of the amino acid chain of major pili and e-pili was found. However, minor pili of *Dv*H had a lower E value and higher query cover percentage, indicating the minor pili shared a certain similarity with e-pili (Table S5). E-pili normally has phenylalanine (F), which aromatic amino acid, at the N terminus, and the majority have leader peptides with less than 12 amino acids (74). Instead of phenylalanine, the minor pili have Tyrosine (Y), which is also an aromatic amino acid. Thus, it is possible that the minor pili that are conductive may contribute to EET of *Dv*H. Being similar to *Dv*H, *D. desulfuricans* produces nanoscale filaments (31). These unidentified filaments were confirmed to be electrically conductive for extracellular electron transfer. *D. desulfuricans* also have both Flp family type IVb pilin and prepilin-type N-terminal cleavage/methylation domain-containing protein according to the identical protein group database in NCBI. Through the above analysis, pili of *Dv*H did not account for 100% of EET, suggesting additional co-occurring direct or indirect mechanisms in this study.

*Dv*H also has flagella, which are composed of flagellin proteins (i.e., DVU1441, DVU2444, and DVU2082) (75), and these proteins do not have sequence similarity with pilin of *Dv*H nor e-pilin. Flagellar and histidine kinase-related proteins were dominant unique peptides in the anode and cathode when compared with dissimilatory sulfate reduction respiration (Fig. 5). It was reported that *Dv*H forms motility halos on solid media that are mediated by flagella-related mechanisms via the CheA3 histidine kinase (35). This indicated that *Dv*H had increased motility or surface attachment in the anodic chamber, which may promote but not conduct EET.

Another type of extracellular structure was reported in previous studies that proposed biosynthesized FeS mediates the electron transport from *Dv*H to the electrode surface (30, 76). For instance, Deng et al. (76) found *Dv*H biosynthesized FeS nanoparticles on the cell membrane in the presence of sulfate and iron as an electron conduit enabling *Dv*H to utilize solid‐state electron donors via direct electron uptake. However, no biosynthesized FeS was observed on the surface of the cathode based on the results of EDS elements analysis, but they were observed as precipitates at the bottom of MFCs. The FeS nanocrystallites may be washed off during the fixation preparation process of SEM. Besides the direct electron transfer conducted by FeS nanoparticles (77), our results indicated that direct contact through pili/filaments may be one of the major routes for direct electron transfer of *Dv*H. Our results showed that indirect electron transfer mechanisms potentially via electron shuttle small molecules, which are flavin-related ones are also happening in *Dv*H. This indicates that *Dv*H can use different possible electron transfer mechanisms to survive in less-than-ideal conditions with solid surfaces as both an electron acceptor and a donor. A comprehensive understanding of *Dv*H’s electron transfer mechanisms can expand its application beyond MFCs to address sulfate-containing water and wastewater in various contexts and applications.

### Conclusions

In this study, we found that *Dv*H was able to attach to the electrodes of MFC and produced different types of filaments connecting the bacterium to the electrode surface; however, direct tests of the conductivity of the pili were inconclusive. During electroactive respiration with DvH on both electrodes in a dual chamber MFC, there was electricity production with a maximum power density of ~0.074 W/m^2^. The ratio of electron donor to acceptor in both chambers and the presence of pili and biofilm were found to be key variables for electricity generation. Untargeted metabolomics profiling showed flavin-based metabolites, which are potential electron shuttles. Taken together, these results indicated that *Dv*H likely has multiple extracellular electron transfer pathways facilitating growth on solid surfaces that can be utilized either as the electron acceptor or donor (Fig. 6). Future work is needed to confirm the activity of this variety of possible mechanisms in contributing to energy production in this metabolically versatile microorganism.

## Data Availability

Data are provided within this manuscript. Proteomics results, as received from the Mass Spectrometry Core Facility in the Biotechnology Center at the University of Wisconsin-Madison, are in Supplemental Tables S6-S9. Metabolomics data have been deposited to the EMBL-EBI MetaboLights database (DOI: https://doi.org/10.1093/nar/gkad1045, PMID:37971328) with the identifier MTBLS11447. Strains will be made available upon request.

## SUPPLEMENTAL MATERIAL

Supplemental figures spectrum.01226-24-s0001.docx Figures S1 to S8.

Supplemental tables spectrum.01226-24-s0002.xlsx Tables S1 to S9.

ASM does not own the copyrights to Supplemental Material that may be linked to, or accessed through, an article. The authors have granted ASM a non-exclusive, world-wide license to publish the Supplemental Material files. Please contact the corresponding author directly for reuse.
