# Supplementary material for: Co-occurrence of direct and indirect extracellular electron transfer mechanisms during electroactive respiration in a dissimilatory sulfate reducing bacterium
Source: Microbiol Spectr. 2024 Dec 5;13(1):e01226-24. doi: 10.1128/spectrum.01226-24 (PMC11705803; doi:10.1128/spectrum.01226-24)
Supplement: Supplemental figures — Figures S1 to S8. [file spectrum.01226-24-s0001.docx]

***Supplemental Materials***

**Co-occurrence of Direct and Indirect Extracellular Electron Transfer Mechanisms during Electroactive Respiration in a Dissimilatory Sulfate Reducing Bacterium**

**Authors**: Liyuan Hou^1,2,3^, Rebecca Cortez^4^, Michael Hagerman^4+^, Zhiqiang Hu^5^, Erica L.-W. Majumder^3*^

^1^ Department of Civil and Environmental Engineering, Utah State University, Logan, UT 84322, USA

^2^ Utah Water Research Laboratory, 1600 Canyon Road, Logan, UT 84321, USA

^3^ Department of Bacteriology, University of Wisconsin-Madison, Madison, WI 53706, USA

^4^ Department of Mechanical Engineering, Union College, Schenectady, NY 12308, USA

^5^ Department of Civil and Environmental Engineering, University of Missouri, Columbia, MO 65201, USA

+ posthumous author

***Correspondence to:**

Dr. Erica Majumder, emajumder@wisc.edu

Department of Bacteriology, University of Wisconsin-Madison, Madison, WI 53706, USA


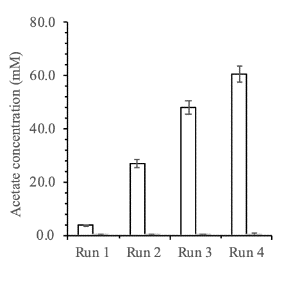

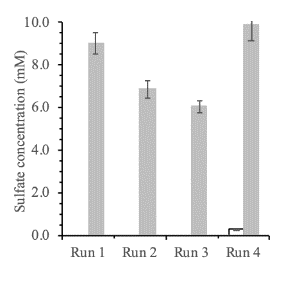

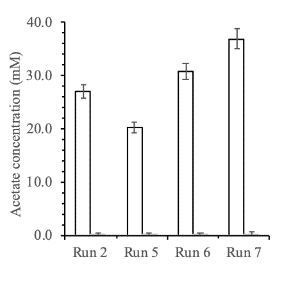

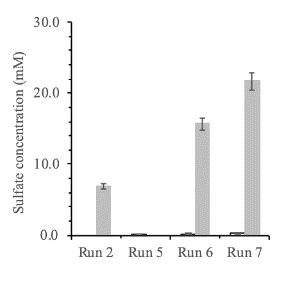


d

c

a

b


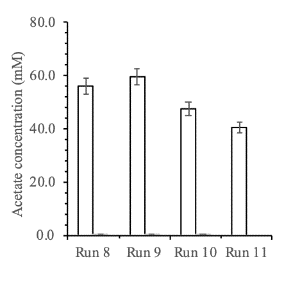

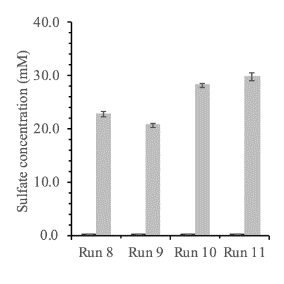


f

e

**Figure S1.** Acetate production (a, c and e) and residual sulfate concentration (b, d and f) and in anode chamber () and cathode chamber (
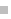
), respectively.


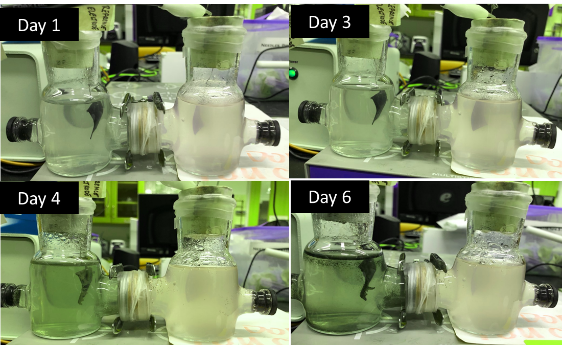


**Figure S2.** Images depicting the changes in biofilms in the MFC for Run 8 at different time points.

**
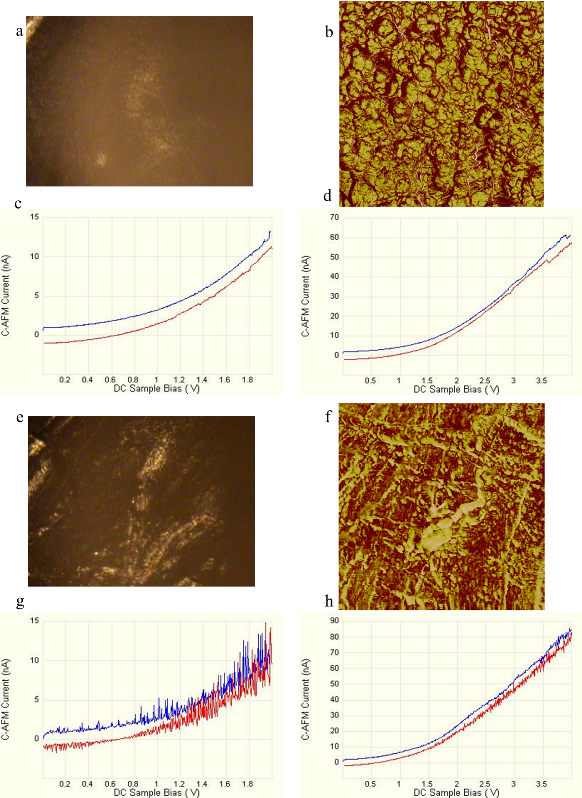
Figure S3.** cAFM results of plain carbon cloths (a - d) and carbon cloths with *Dv*H biofilm (e - h). (a and e: optical micrographs; b and f: AFM phase images where the black to white grayscale is 45°; c and g: the variation of currents when voltage (0~2 V) was applied to the area near the center of the image; d and h: the variation of currents when voltage (0~4 V) was applied. For the current-voltage curves, blue lines represent the voltage sweep from zero to the maximum voltage, while red lines represent the voltage sweep from maximum voltage back to zero.) Below are larger views of S3b and d showing the scale bar.


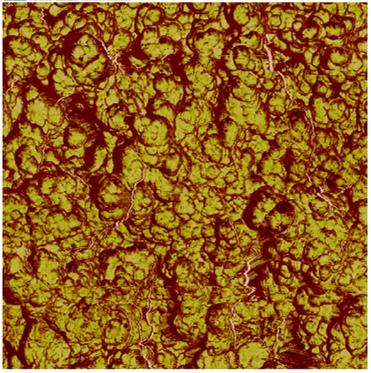


**500 nm**


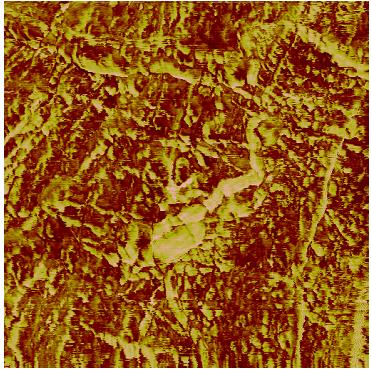


**500 nm**


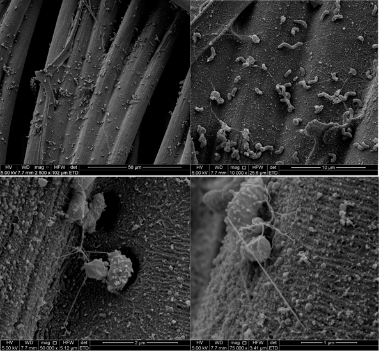


a


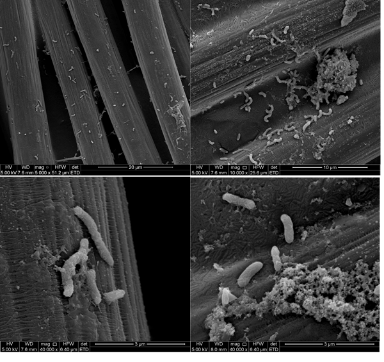


b


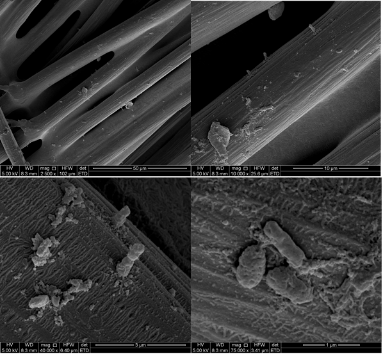


c

e

d


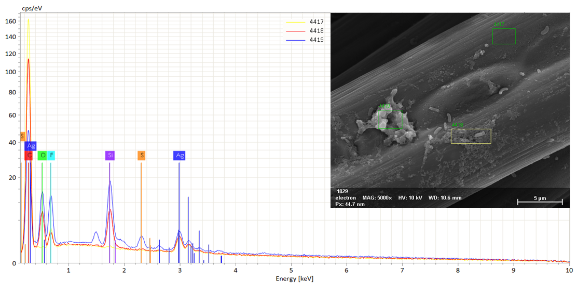


**Figure S4** Additional SEM images of wild type *Dv*H JWT700 (a), *Dv*H JW3422 (pili lacking mutant) (b) and *Dv*H JWT716 (biofilm lacking mutant) (c) and Identification of elemental composition of small areas on the surface of carbon cloths for wild type *Dv*H JWT700 (d) and *Dv*H JW3422 (pili lacking mutant) (e).


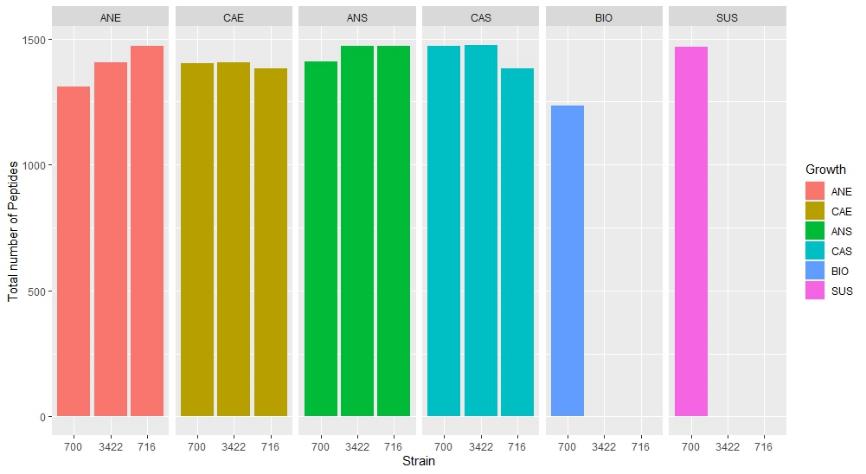
**Figure S5** The number of peptides from *Dv*H JWT700, *Dv*H JW3422 and *Dv*H JWT716 across various electroactive respiration modes and without electroactive repatriation modes.


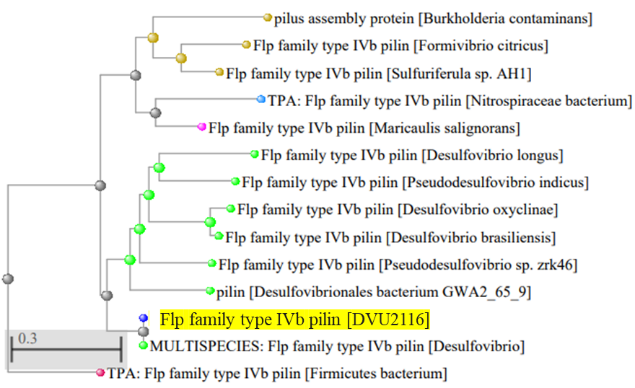


**Figure S6** The tree was produced in Unipro UGENE using BLAST pairwise alignments between the pili of *Dv*H (DVU2116) and other flp family type IVb pili.


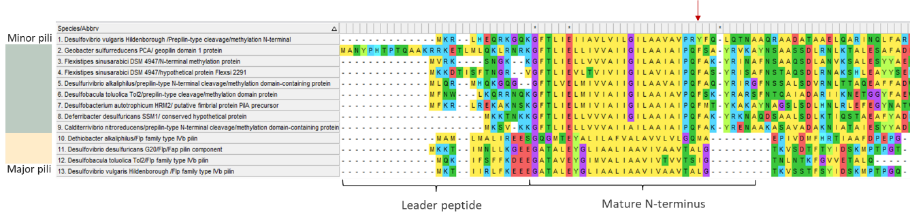


**Figure S7** Protein **s**equence alignment produced in Unipro UGENE between the major and minor pili of *Dv*H and pili produced by other bacteria with documented extracellular electron transfer. The green bar indicates e-pili while the yellow bar indicates type IVb pili that are not conductive.

a


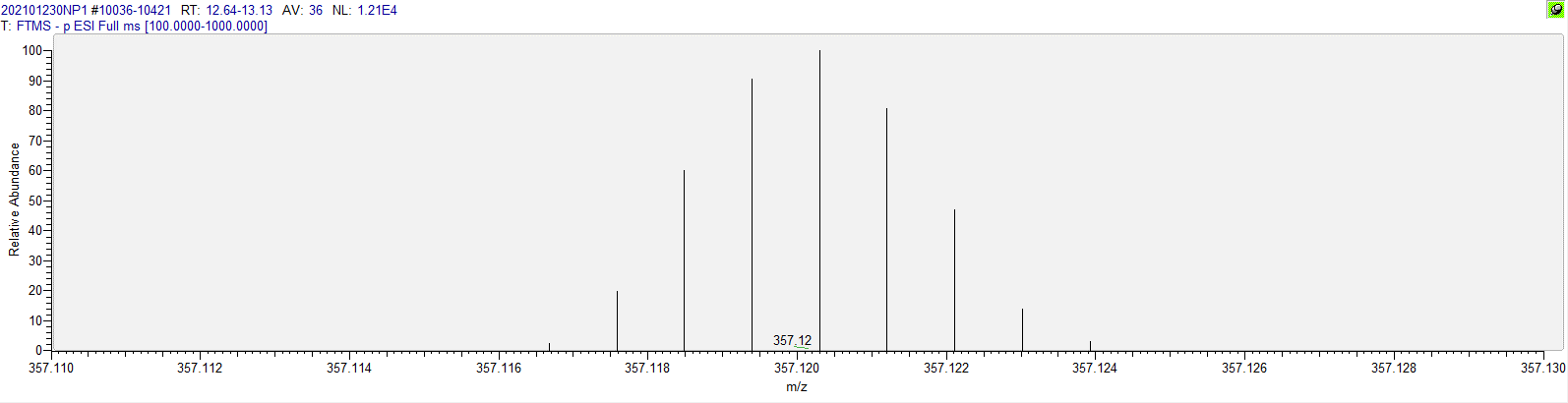


b


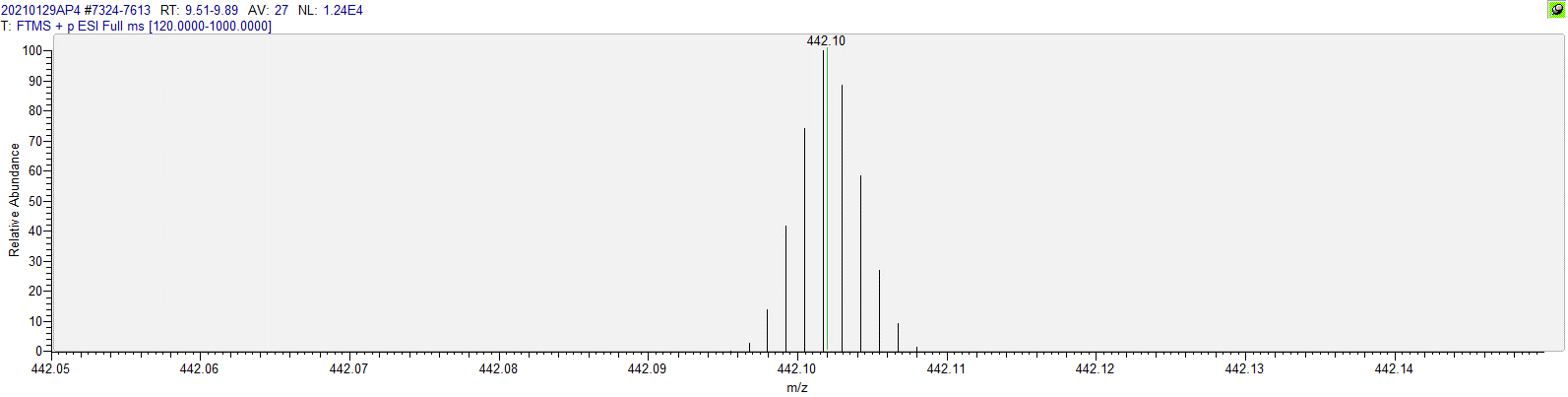


c


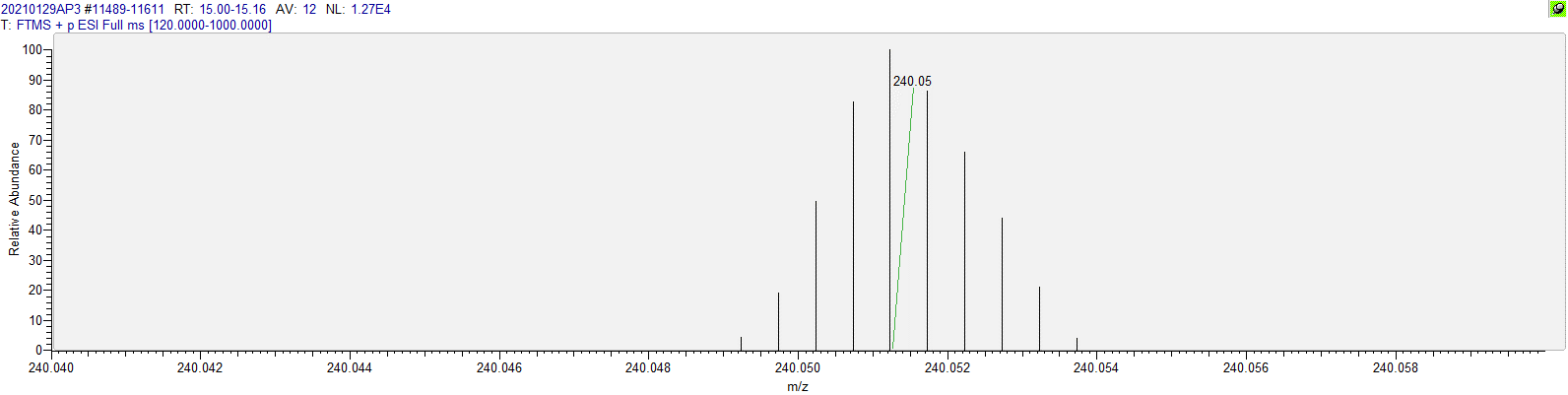


**Figure S8** MS/MS spectra of peaks identified as riboflavin (a), FMN (b), and FMNH_2_ (c).
